# Supplementary material for: Dependency on host vitamin B12 has shaped Mycobacterium tuberculosis Complex evolution
Source: Nat Commun. 2024 Mar 9;15:2161. doi: 10.1038/s41467-024-46449-8 (PMC10924821; doi:10.1038/s41467-024-46449-8)
Supplement: Supplementary file 1 — Supplementary Information [file 41467_2024_46449_MOESM1_ESM.pdf]

SUPPLEMENTARY INFORMATION

Dependency on host vitamin B12 has shaped *Mycobacterium tuberculosis* Complex evolution

Elena Campos-Pardos [1,2], Santiago Uranga [1,2], Ana Picó [1,2], Ana Belén Gómez [1,2],

Jesús Gonzalo-Asensio [1,2,*]

[1] Grupo de Genética de Micobacterias, Departamento de Microbiología y Medicina Preventiva. Facultad de Medicina, Universidad de Zaragoza, IIS Aragón, Zaragoza, Spain

[2] CIBER Enfermedades Respiratorias, Instituto de Salud Carlos III, Madrid, Spain

* Corresponding author: jagonzal@unizar.es

Index:

Supplementary Figures page 2

Supplementary Notes page 25

Supplementary Tables page 31

Figure S1. Sanger sequencing chromatograms showing B12 mutations in clinical isolates belonging to specific MTBC lineages. The upper chromatograms represent the sequence of the specified *cob* gene in *M. tuberculosis* H37Rv, and the lower chromatograms depicts sequences in clinical isolates from MTBC lineages. Red boxes indicate the nucleotide(s) affected by the genetic polymorphisms.

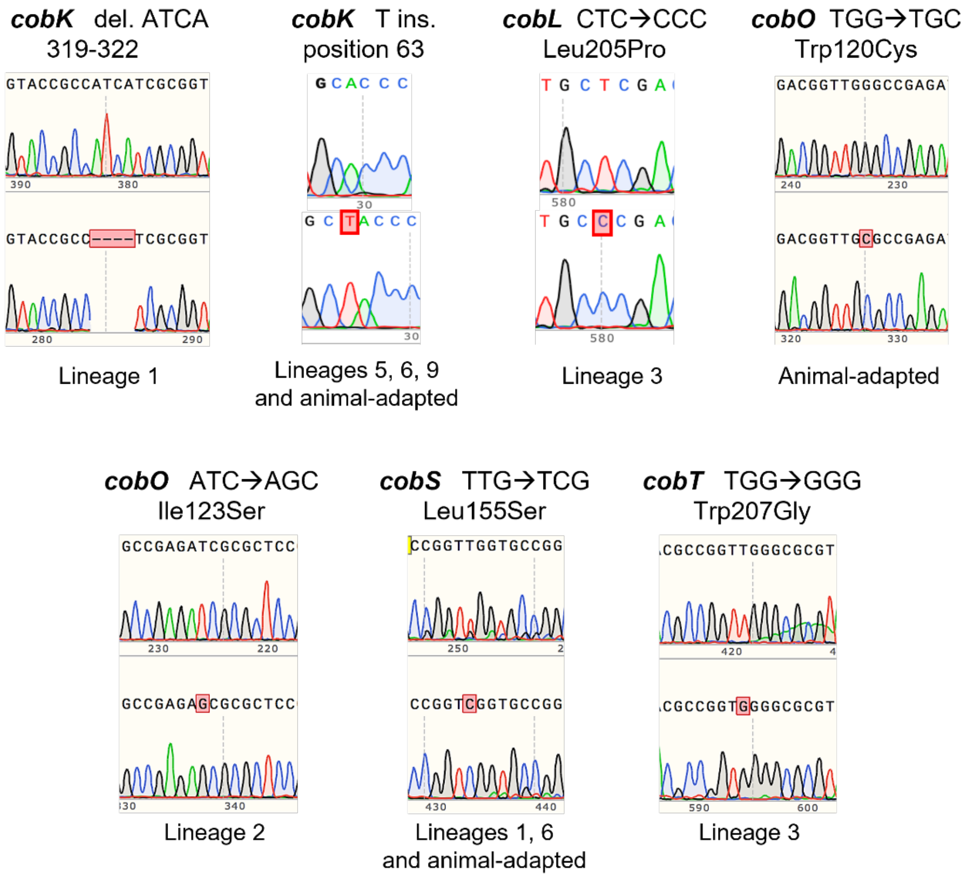

Figure S2. Uptake of exogenous cyanocobalamin (CN) and adenosyl cobalamin (Ado) by MTBC strains cultured until stationary growth-phase. Bars represent the mean and standard deviation (SD) from at least three biological replicates.

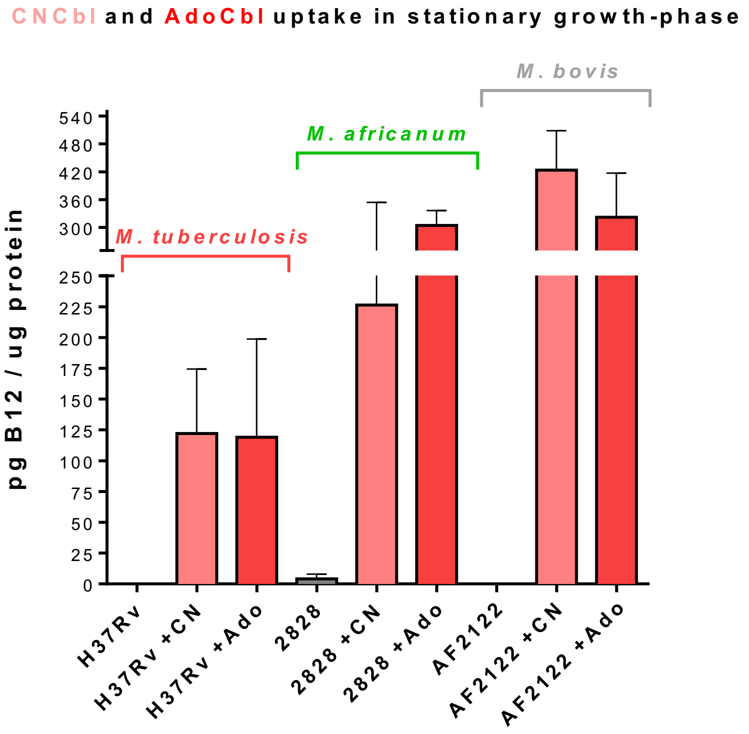

Figure S3. Characterization of the *in vitro* growth of *M. tuberculosis* H37Rv in the presence or absence of vitamin B12. (A) OD=600 nm measures in liquid cultures of H37Rv supplemented with B12 (red) compared to cultures without B12 supplementation (grey). **(B)** Colony forming units (CFUs) per mL grown on 7H10-ADC plates supplemented (red) or not (grey) with B12. A standard, non-supplemented, liquid culture of *M. tuberculosis* H37Rv was used as inoculum for solid plates.

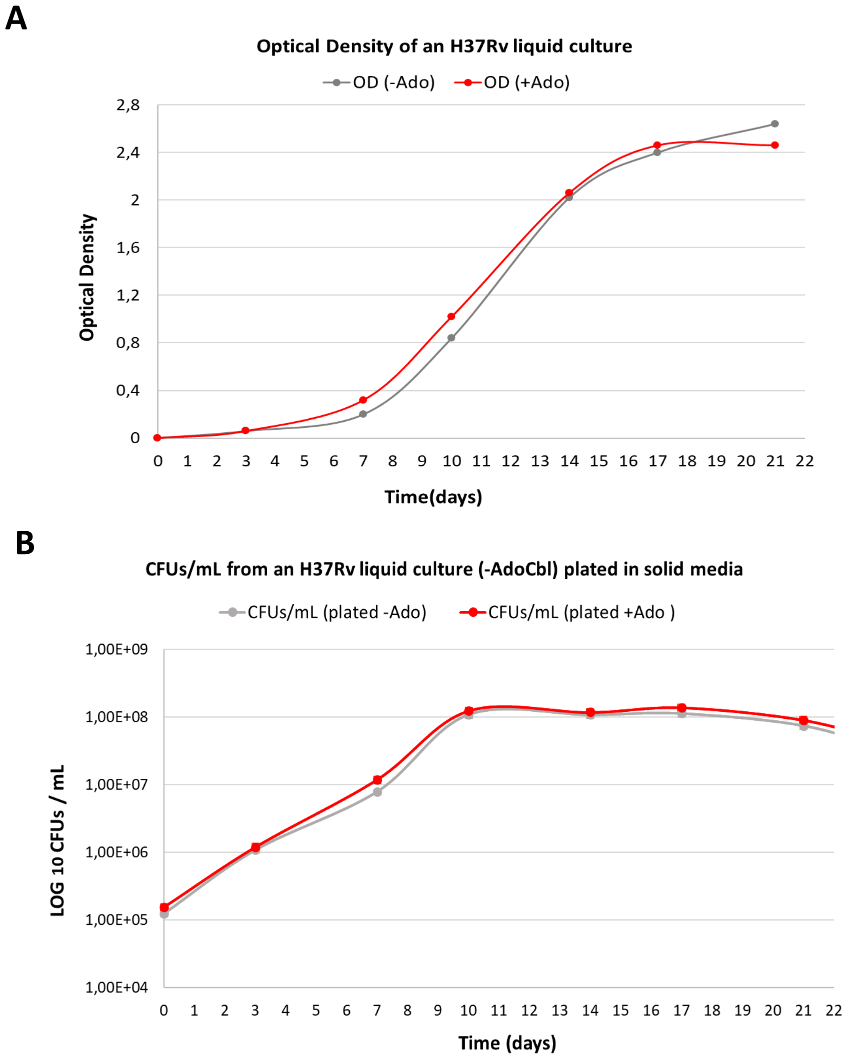

Figure S4. Development of the *M. canettii* C59 infection model in immunocompromised SCID mice and optimization of the bacterial infection inoculum. Survival rates of the infected mice with the different infection doses of *M. canettii* C59 are indicated by different shades of blue. Survival of mice infected with *M. tuberculosis* H37Rv is shown in red, which was used as control strain.

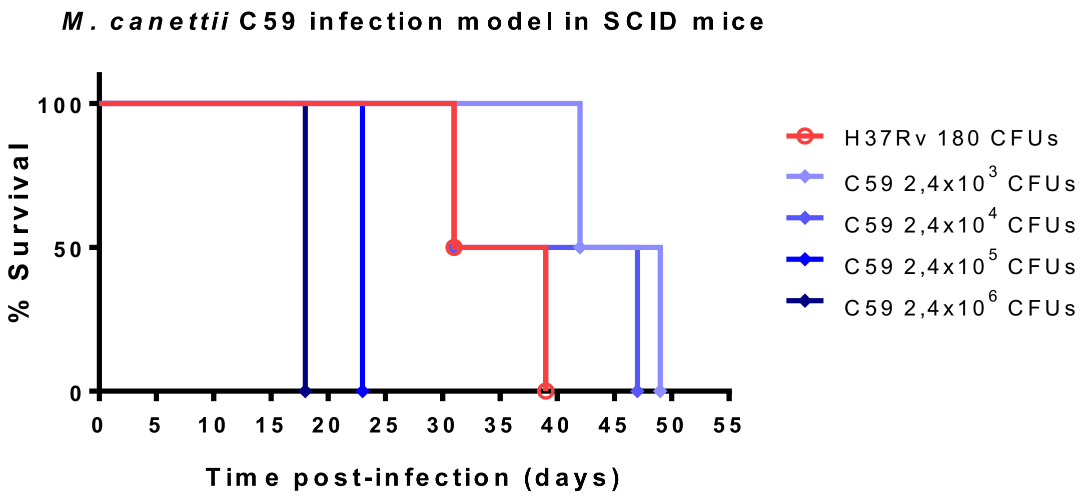

Figure S5. Serum B12-levels in B12 anemic mice at the endpoint of infections with wild type strains of *M. tuberculosis* and *M. canettii*. Graph data are mean ± SEM of at least three biological replicates. Statistical analysis was performed using two-way ANOVA followed by Sidak's post-test. Asterisks indicate the following p values: ** 0.01 > p > 0.001; **** 0.0001 > p.

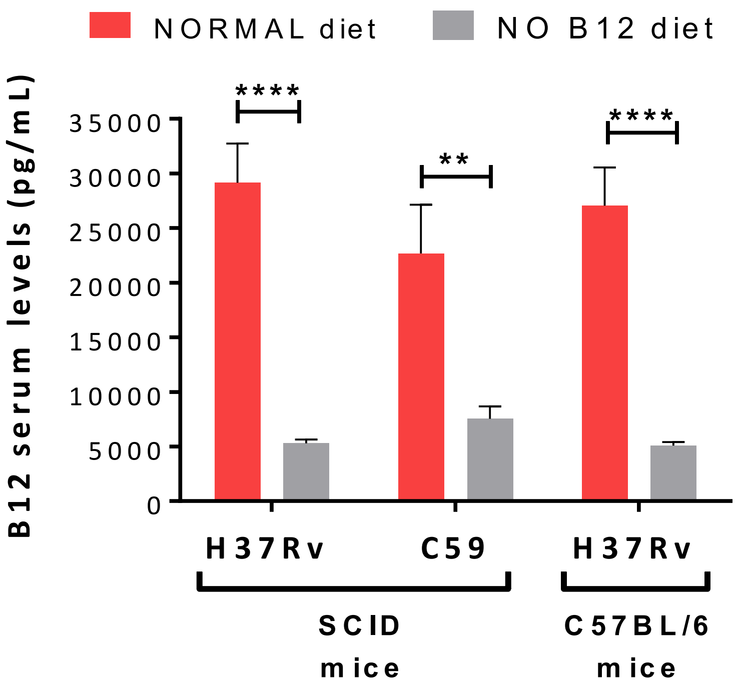

Figure S6. Body weight evolution of SCID mice until reaching criteria to determine the humane endpoint. Upper graphs show the evolution of the mean body weight of anemic (gray lines) and control (red lines) mice infected with wild type strains of *M. tuberculosis* H37Rv (A) or *M. canettii* C59 (B). Lower graphs indicate the individual evolution of the body weight of each mice infected with the corresponding strains.

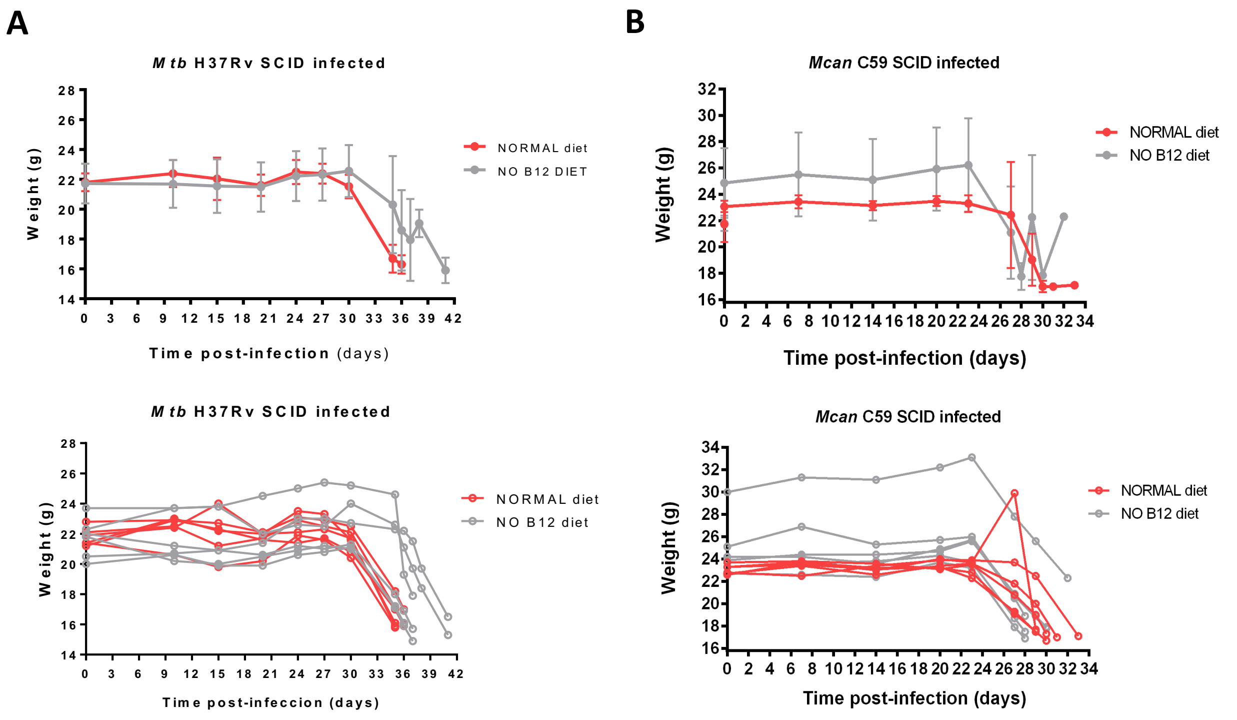

Figure S7. Experimental replicates of C57BL/6 mice infections. Results show organ CFUs from animals fed with control, or B12-deficient diets, and infected with *M. tuberculosis* H37Rv, *M. canettii* C59, *M. tuberculosis* Δ*metE* and Δ*metH* mutants, and the Δ*metH* Pr_{Ag85a}*metE*::Kan complemented strain.

A. Bacterial loads in the lungs and spleens of control, and B12-deficient, C57BL/6 mice after 4 weeks infected with *M. tuberculosis* H37Rv or *M. canettii* C59. Data represent mean ± SD of each mice group, and statistical analysis was performed using Mann-Whitney test for each strain. P values are indicated as follows: ** 0.01 > p > 0.001; ns: not significant, p ≥ 0.05.

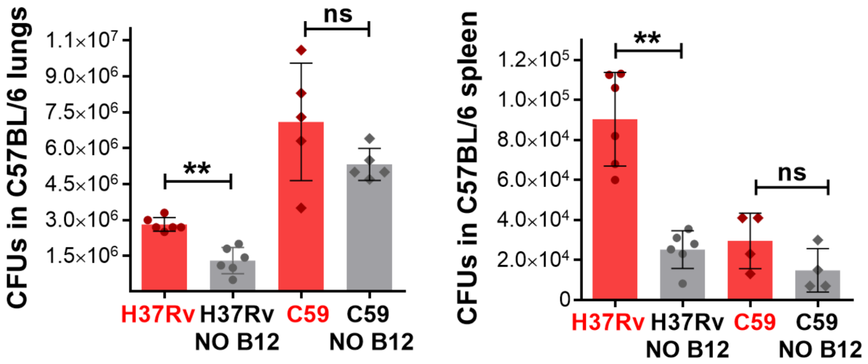

B. Bacterial loads in the lungs of control, and B12-deficient, C57BL/6 mice after 4 weeks infected with *M. tuberculosis* H37Rv and its Δ*metE* mutant. Data are mean ± SD of at least four replicates. Statistical analysis was performed using Mann-Whitney test for each strain. P values are indicated as follows: * 0.05 > p > 0.01; ** 0.01 > p > 0.001.

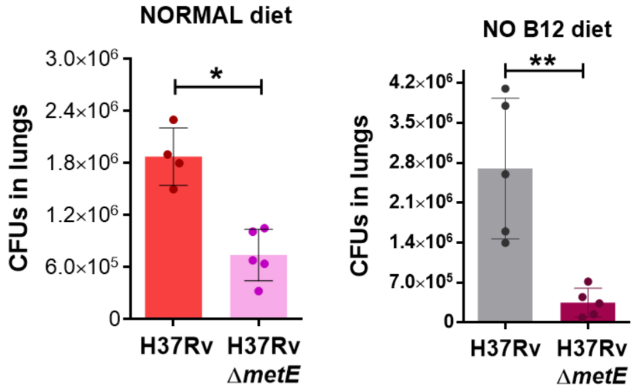

C. Bacterial loads in the lungs of control, and B12-deficient, C57BL/6 mice after 4 weeks infected with *M. tuberculosis* H37Rv and its Δ*metH* mutant. Data are mean ± SD of at least five replicates. Statistical analysis was performed using Mann-Whitney test for each strain. P values are indicated as follows: * 0.05 > p > 0.01; ns: not significant, p ≥ 0.05.

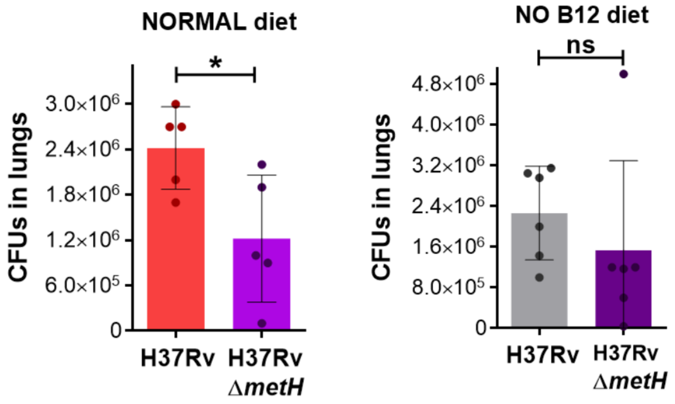

D. Bacterial loads in the lungs of control, and B12-deficient C57BL/6 mice after 4 weeks infected with *M. tuberculosis* H37Rv, the Δ*metH* knockout, or the Δ*metH* Pr_{Ag85a}*metE*::Kan Data are mean ± SD of at least five replicates. Statistical analysis was performed using Mann-Whitney test for each strain. P values indicated as ns means not significant.

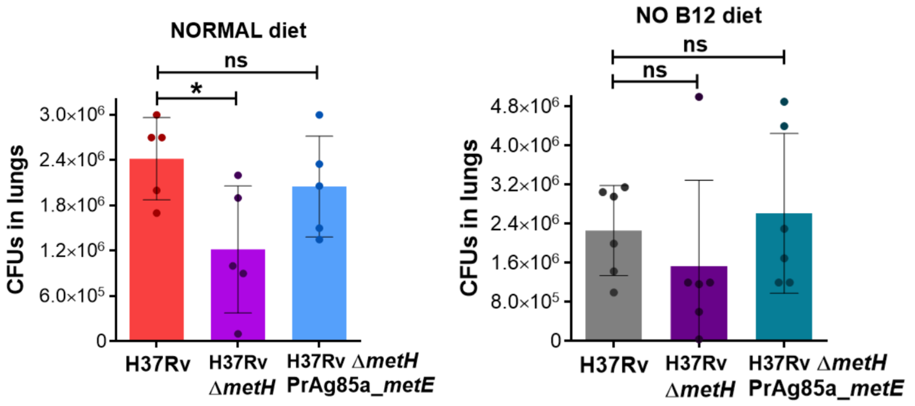

Figure S8. Construction of a *M. canettii* C59 mutant defective in B12 production results in increased survival of immunocompromised SCID mice and decreased lung bacterial burden in immunocompetent C57BL/6 mice. (A) Validation of the decreased B12-production in the C59 *M. canettii* Δ*cobMK* mutant. Measures of B12 in bacterial extracts from *in vitro* cultures of the mutant and the wild type strain. **(B)** Survival rates of SCID mice fed with a B12-deficient diet infected with the *M. canettii* Δ*cobMK* mutant (yellow line) compared to its wild type strain (gray line). P value is indicated as follows: ** $0.01 > p > 0.001$ [Log-rank (Mantel-Cox) test]. **(C)** CFUs counts in lungs of B12-anemic C57BL/6 mice infected with the aforementioned strains. Data are mean ± SD of at least five replicates. Statistical analysis was performed using Mann-Whitney test. P value is indicated as follows: ** $0.01 > p > 0.001$.

A

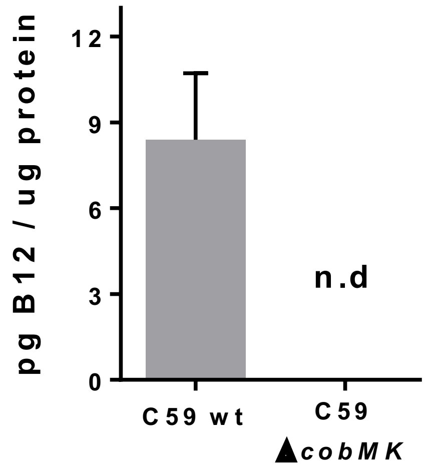

B

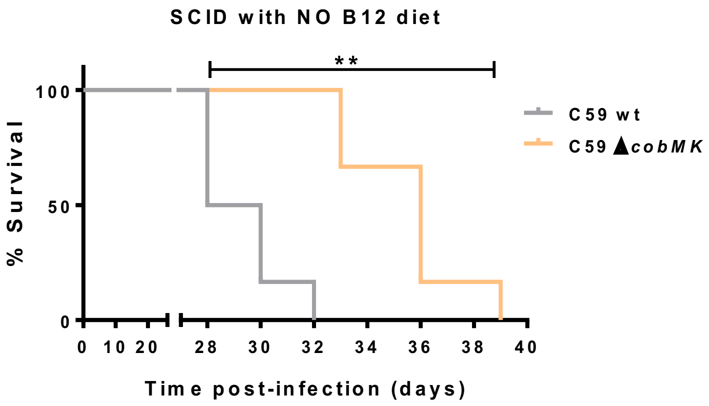

C

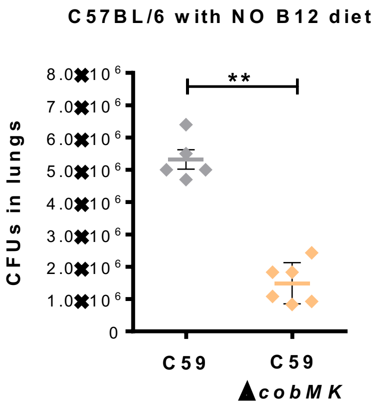

Figure S9. Transcriptional profiles of genes from the "core B12 regulon". (A) RNA-seq profiles of the PPE2-*cobQ1*-*cobU* region from *M. tuberculosis* H37Rv in response to exogenous B12 supplementation (light blue) relative to the experimental control without B12 (dark blue). **(B)** qRT-PCR of Rv1129c (*prpR*), *prpD*, *metE, PPE2, cobQ1* and *sigA* in *M. tuberculosis* H37Rv cultures grown with or without B12. Relative quantity refers to the differential expression of the selected genes in the presence of B12 in comparison with its expression in the absence of B12. Each gene was normalized against *sigA* expression in each sample. Graphs represents mean ± SD from three biological replicates.

A

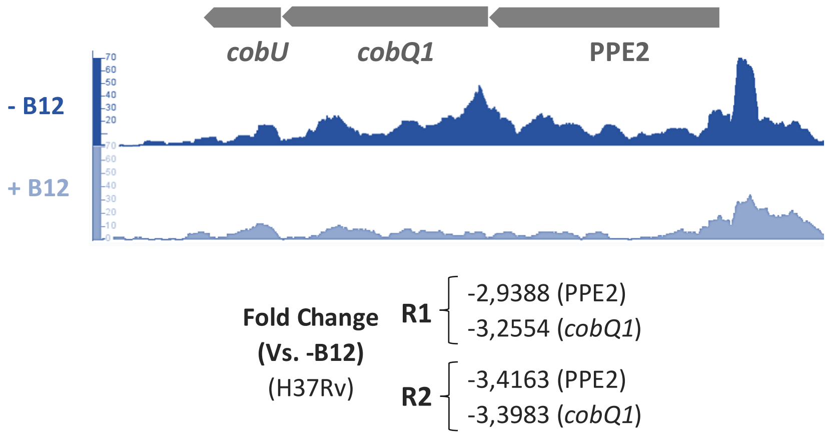

B

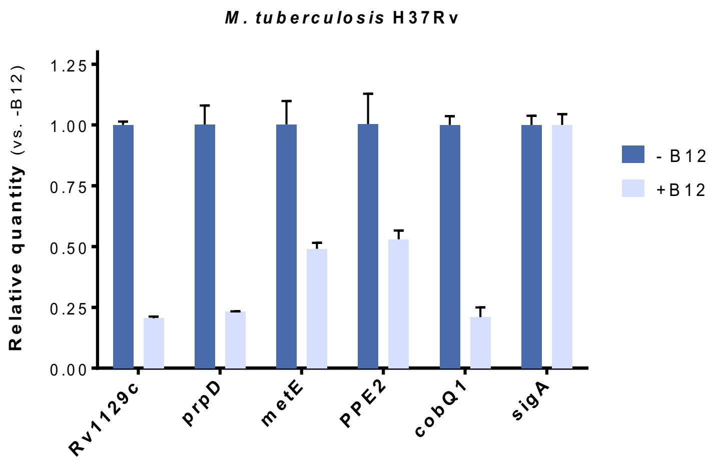

Figure S10. SRM/MS profiles of selected peptides from MetE, PrpC and PrpD proteins of *M. tuberculosis* H37Rv and GC1237 cultures grown with or withour B12.

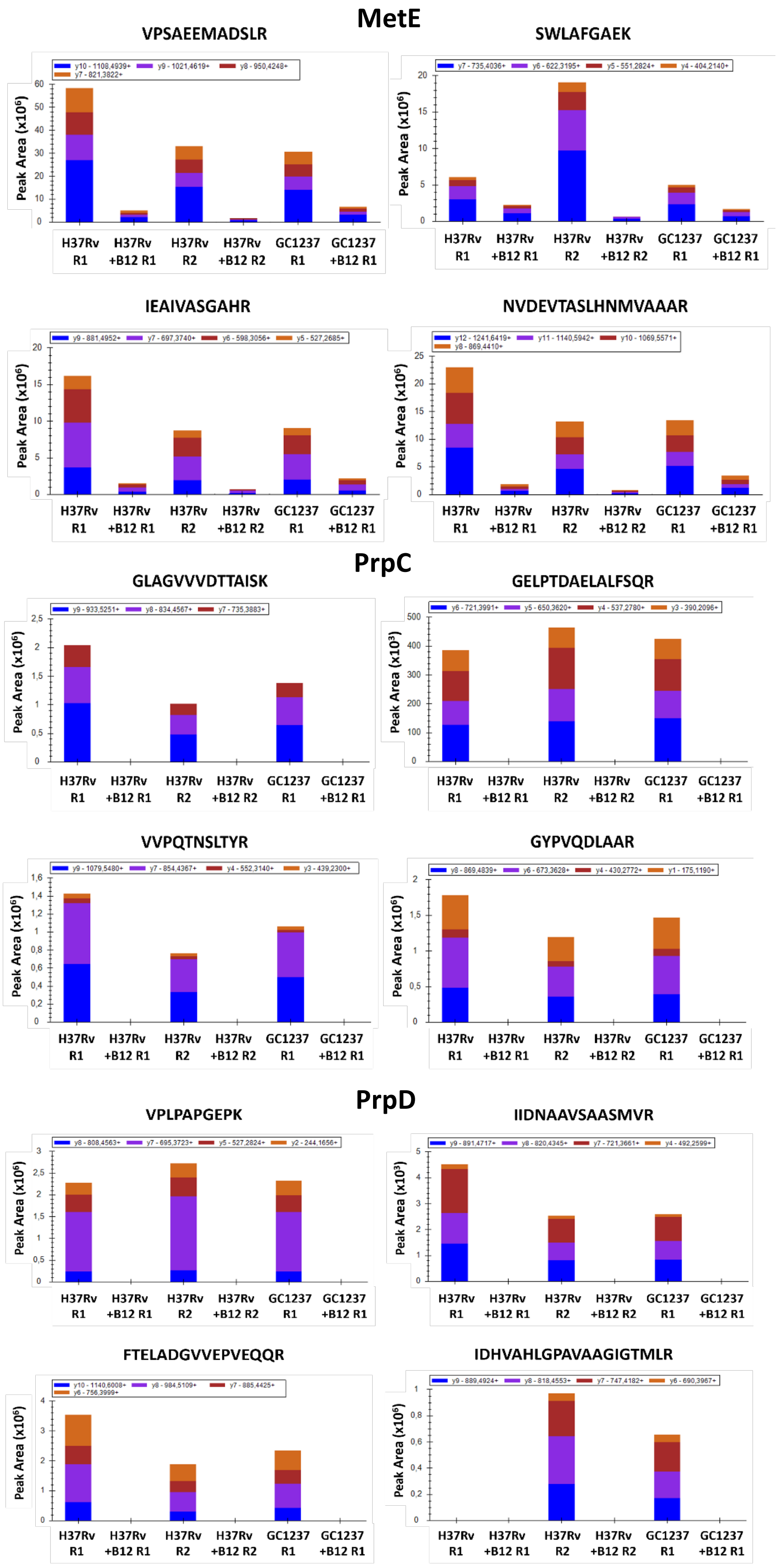

Figure S11. Dose-dependent effect of B12 over MetH and MetE. A) Determination of the B12 concentration able to support MetH functionality. A *M. tuberculosis* Δ*metE* mutant was grown in the presence of a B12 concentration ranging from 10-0.01 μg/mL Adenosylcobalamin. It is observed that 0.01 μg/mL still allows *M. tuberculosis* Δ*metE* to grow albeit at lower rates than at higher B12 concentrations. B) Determination of the B12 concentration able to inhibit the *metE* riboswitch. A *M. tuberculosis* Δ*metH* mutant was grown in the presence of a B12 concentration ranging from 100-0.01 μg/mL Adenosylcobalamin. It is appreciated that at 0.01 μg/mL B12 the mutant fails to recover growth, indicative of *metE* inhibition by the B12-dependent riboswitch.

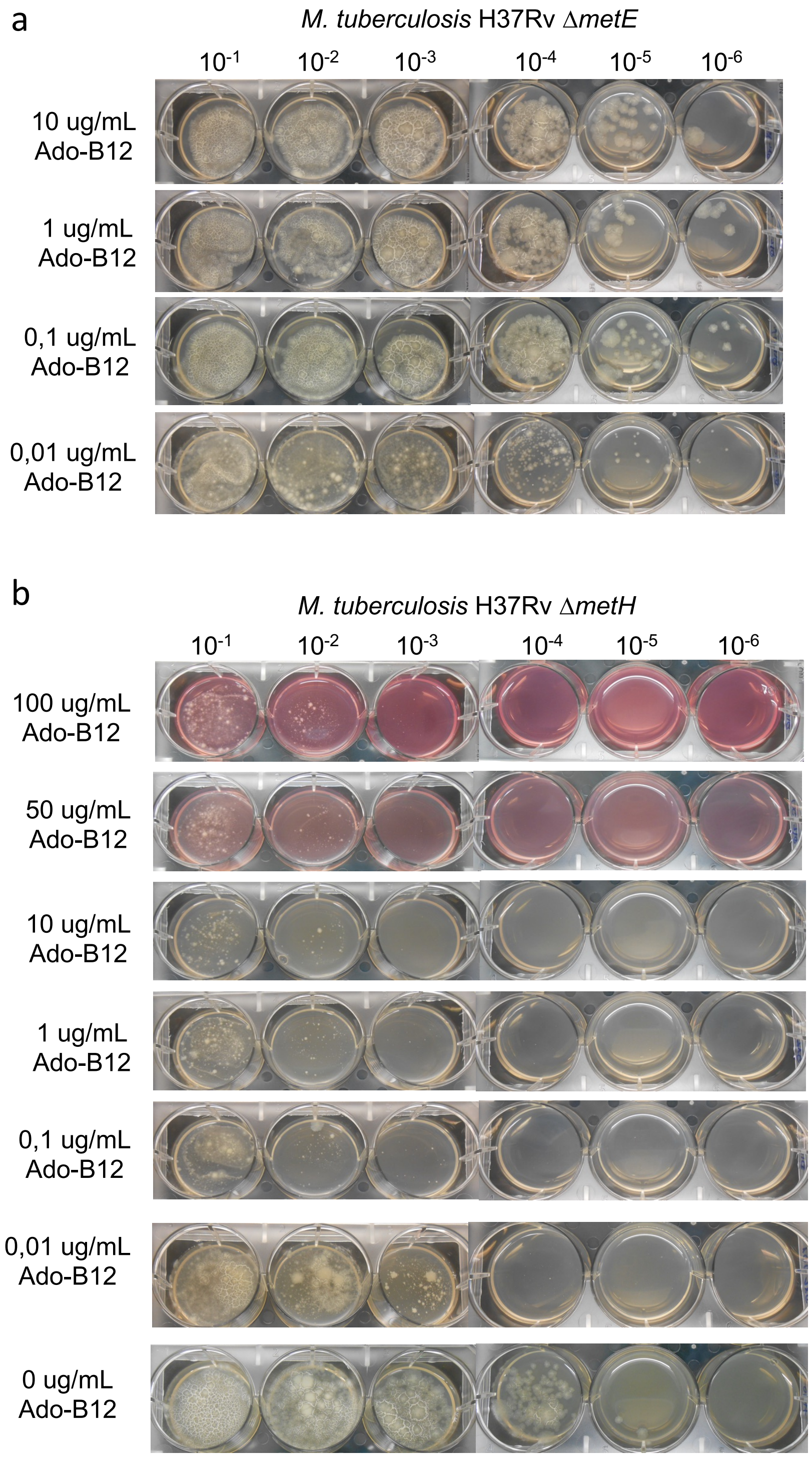

Figure S12. Growth of *M. smegmatis* and *M. canettii* derivatives in the presence of B12 and/or L-methionine. (A) The upper panel shows *in vitro* growth of a *M. smegmatis* Δ*cobLMK* mutant defective in B12 biosynthesis in plates supplemented with or without B12. The lower panels depict growth on solid plates of a *M. smegmatis* Δ*cobLMK* Δ*metE* mutant with or without B12 and/or L-methionine. Note that the *M. smegmatis* Δ*cobLMK* Δ*metE* mutant is unable to grow without B12 since this cofactor is required for the proper L-methionine synthesis by MetH. Also note that this phenotype is successfully rescued by the addition of exogenous L-methionine, indicative that *M. smegmatis* is able to uptake this aminoacid. **(B)** These panels show growth of *M. canettii* and its *metE* and *metH* mutants supplemented with B12 and/or L-methionine. Note that *M. canettii* Δ*metE* mutant fail to grow to wild type levels without B12 due to inefficient synthesis of L-methionine by MetH. Supplementation with exogenous L-methionine restores wild type growth indicative of a L-methionine uptake mechanism in *M. canettii*. Also note that the *M. canettii* Δ*metH* mutant is unable to grow when B12 is present due to the inhibition of the B12-dependent *metE* riboswitch. This growth defect is rescued by L-methionine supplementation, confirming the presence of the L-methionine transport in *M. canettii*.

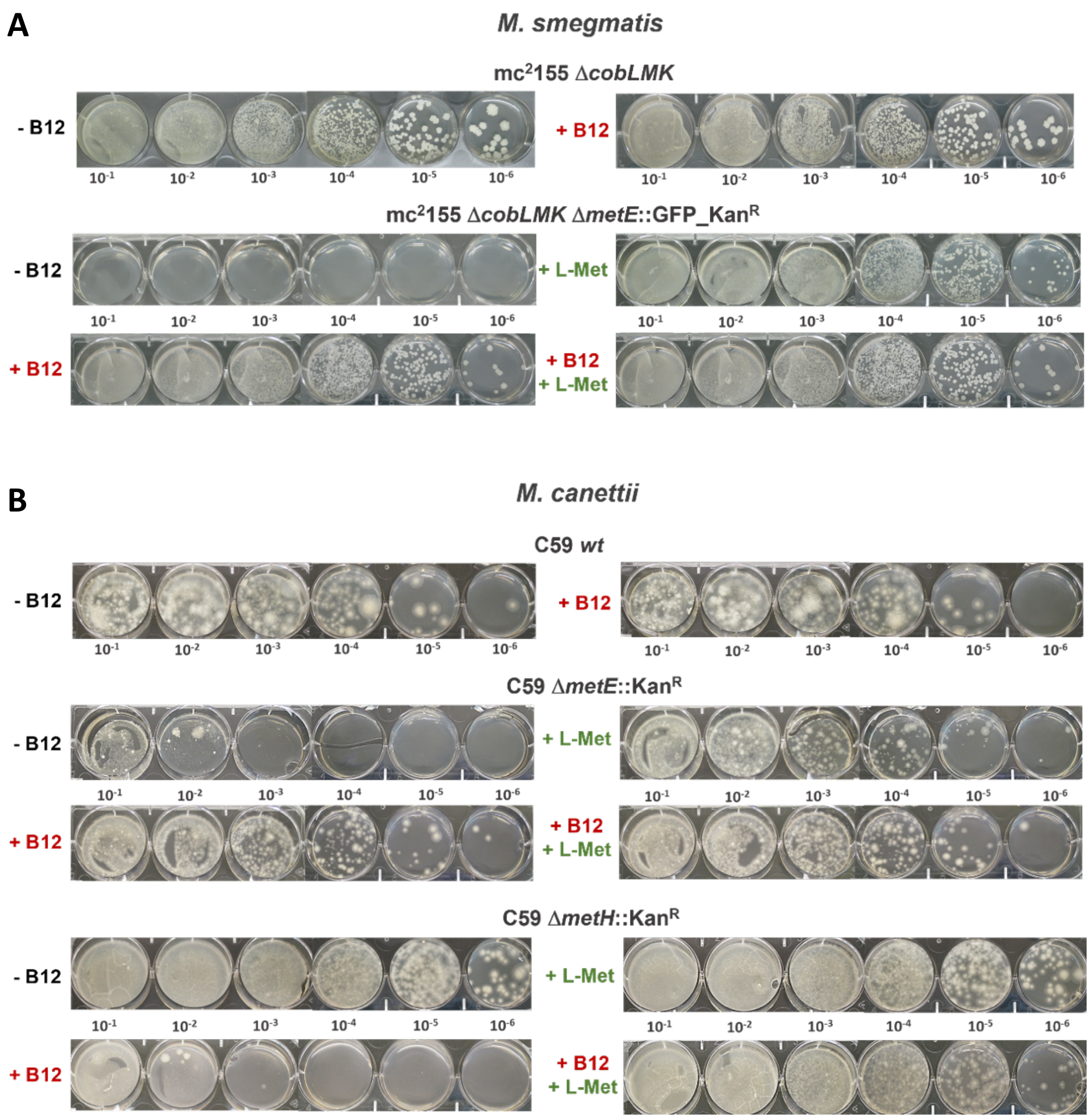

Figure S13. Growth of *M. tuberculosis* ∆*metE* and ∆*metH* mutants in the presence of B12 and/or L-methionine. A) and B) These panels show growth of the *M. tuberculosis* ∆*metE* mutant supplemented with different concentrations of L-methionine. Cultures with B12 are used as growth controls. A) The *M. tuberculosis* ∆*metE* mutant show equivalent grow compared to the control in liquid media supplemented with L-methionine, indicative of L-methionine assimilation during planktonic growth. B) The *M. tuberculosis* ∆*metE* mutant fails to grow to wild type levels without B12 irrespective of the presence of L-methionine on agar plates. These results indicate that assimilation of L-methionine by *M. tuberculosis* occurs during planktonic growth, but not when growing on solid media. C) and D) Growth of the *M. tuberculosis* ∆*metH* mutant supplemented with different concentrations of L-methionine and B12. Cultures without B12 are used as growth controls. C) The *M. tuberculosis* ∆*metH* mutant show equivalent grow compared to the control in liquid media supplemented with L-methionine and B12, indicative of L-methionine assimilation during planktonic growth. D) The *M. tuberculosis* ∆*metH* mutant does not restore wild type grow independently of the presence of L-methionine on agar plates. Again, results support that assimilation of L-methionine by *M. tuberculosis* preferentially occurs during planktonic growth.

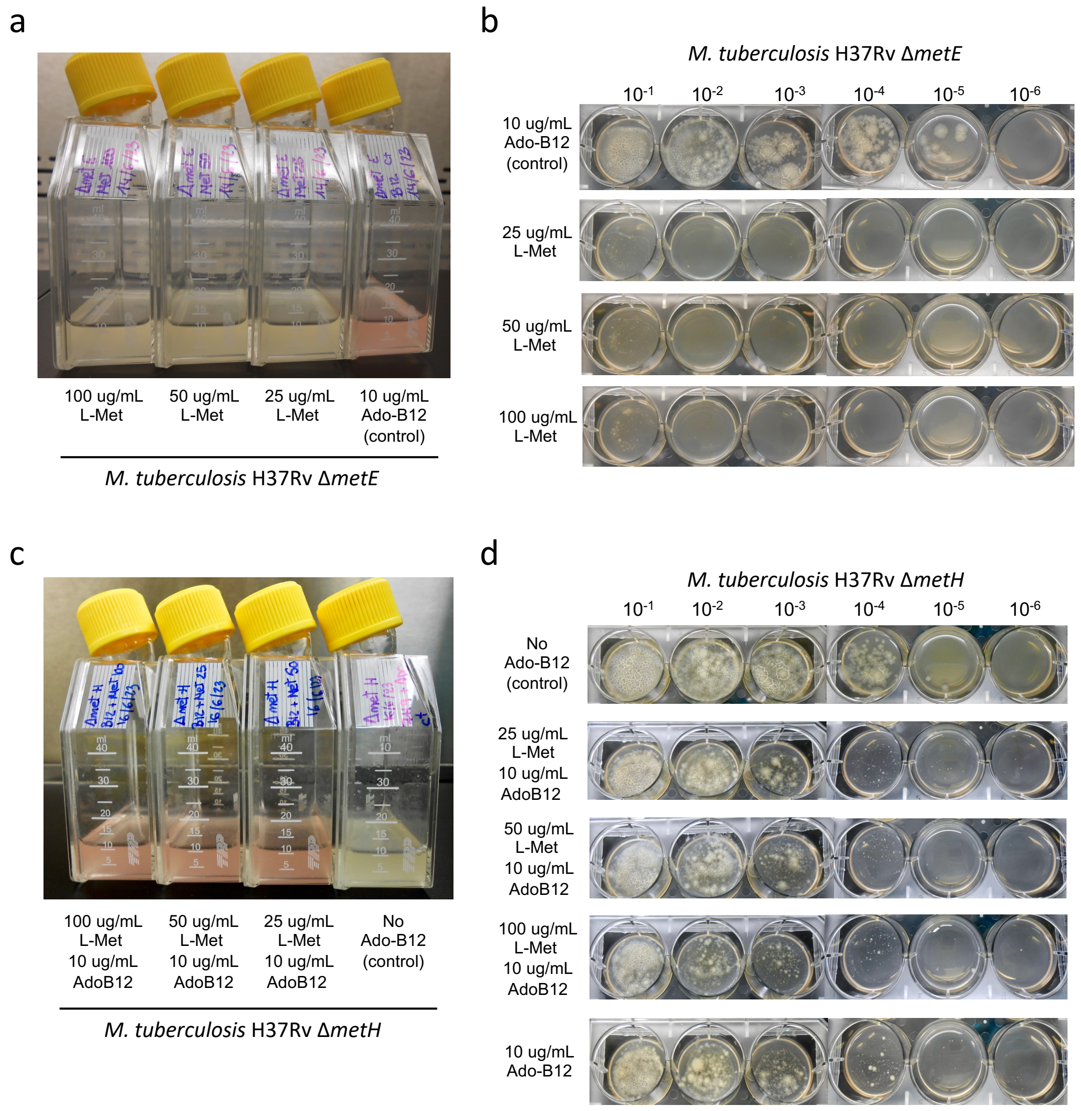

Figure S14. Mutations in the B12 riboswitch upstream the *metE* gene arosen after cultivation of the *M. tuberculosis* Δ*metH* mutant in the presence of B12. (A) Colonies grown on a 7H10-ADC plate supplemented with adenosylcobalamin after plating the *M. tuberculosis* Δ*metH* mutant. **(B)** Sanger chromatograms from colonies described in panel A indicating polymorphisms (red boxes) relative to the *M. tuberculosis* H37Rv reference sequence in different locations of the *metE* riboswitch. Positions refer to nucleotides immediately upstream of the *metE* initiation codon.

A

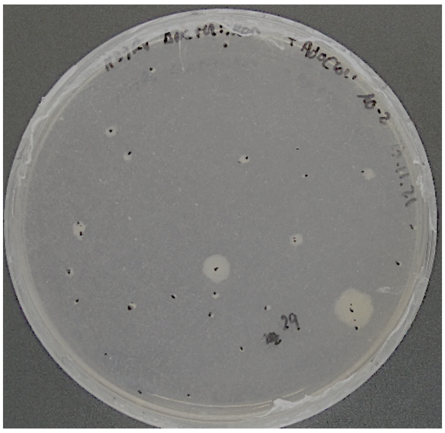

B

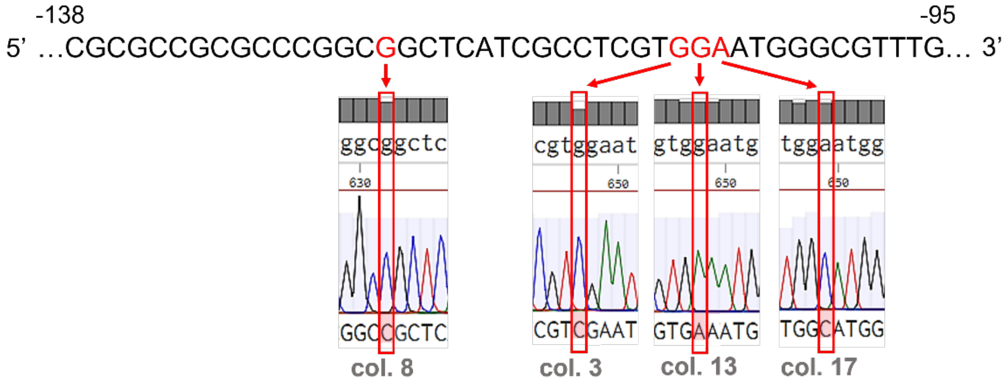

Figure S15. Construction and PCR verification of *M. tuberculosis*, *M. canettii* and *M. smegmatis* mutant and recombinant strains used in this study. For clarity, description of each mutant/recombinant strain will be provided in each panel.

A. Construction of a *M. canettii* Δ*cobMK* mutant

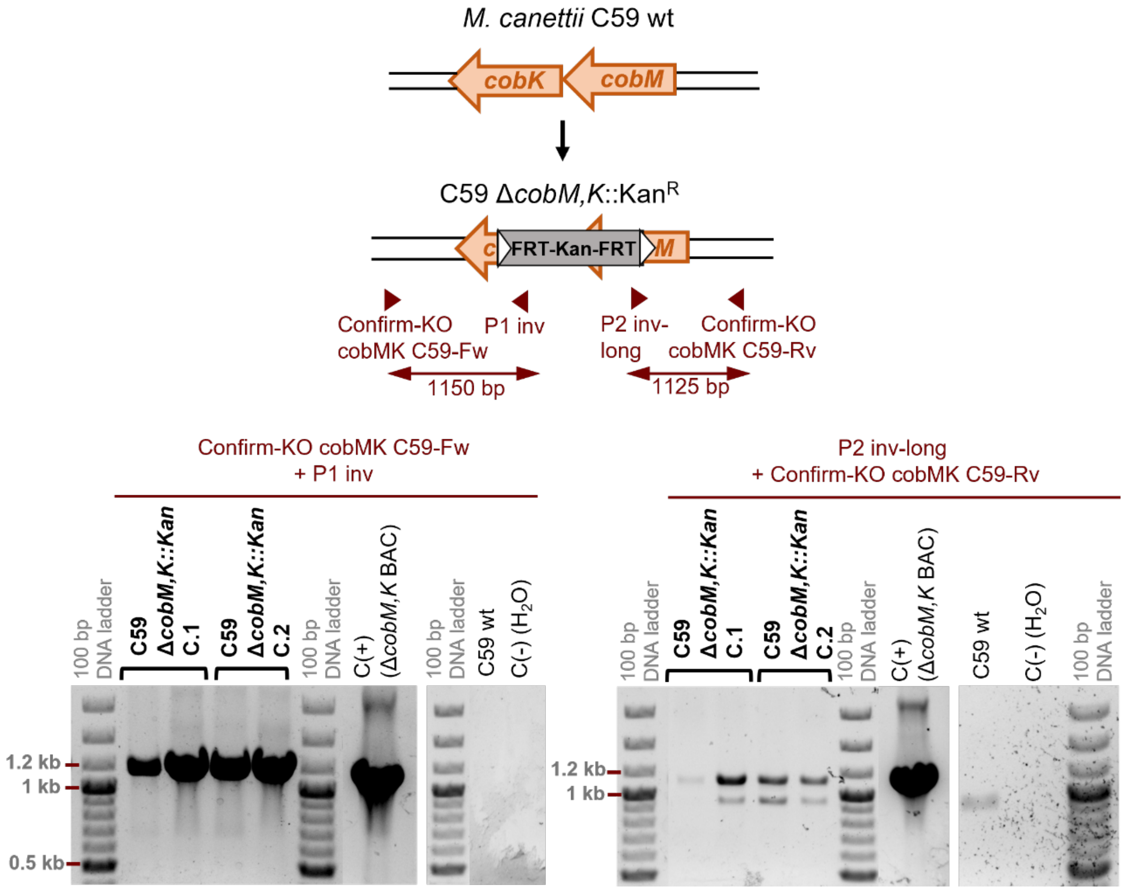

B. Construction of a *M. tuberculosis* Δ*metE* mutant

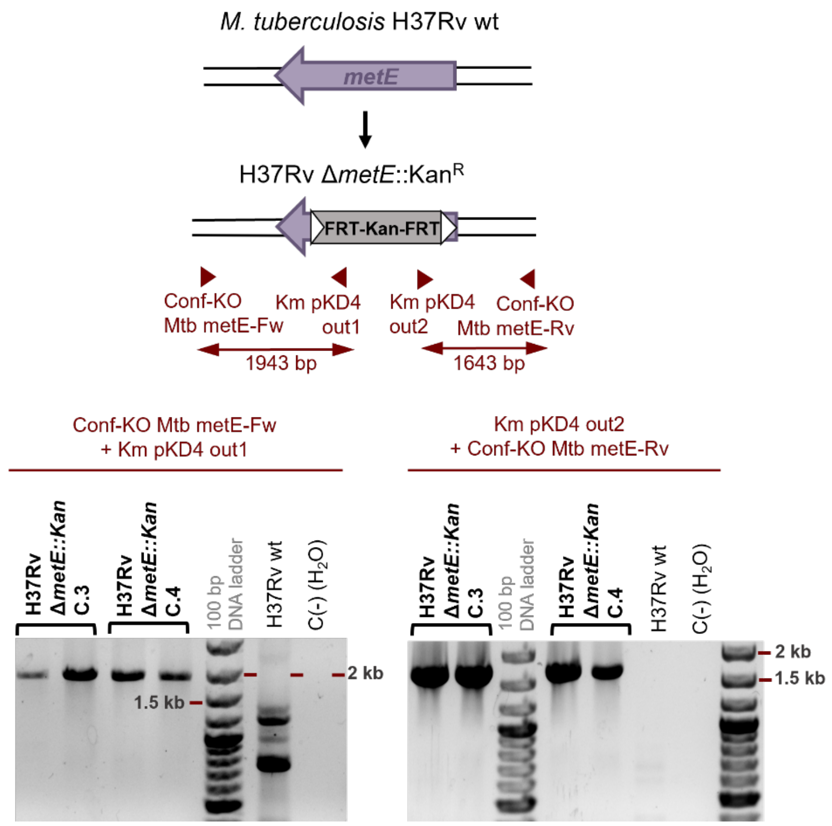

C. Construction of a *M. tuberculosis* Δ*metH* mutant

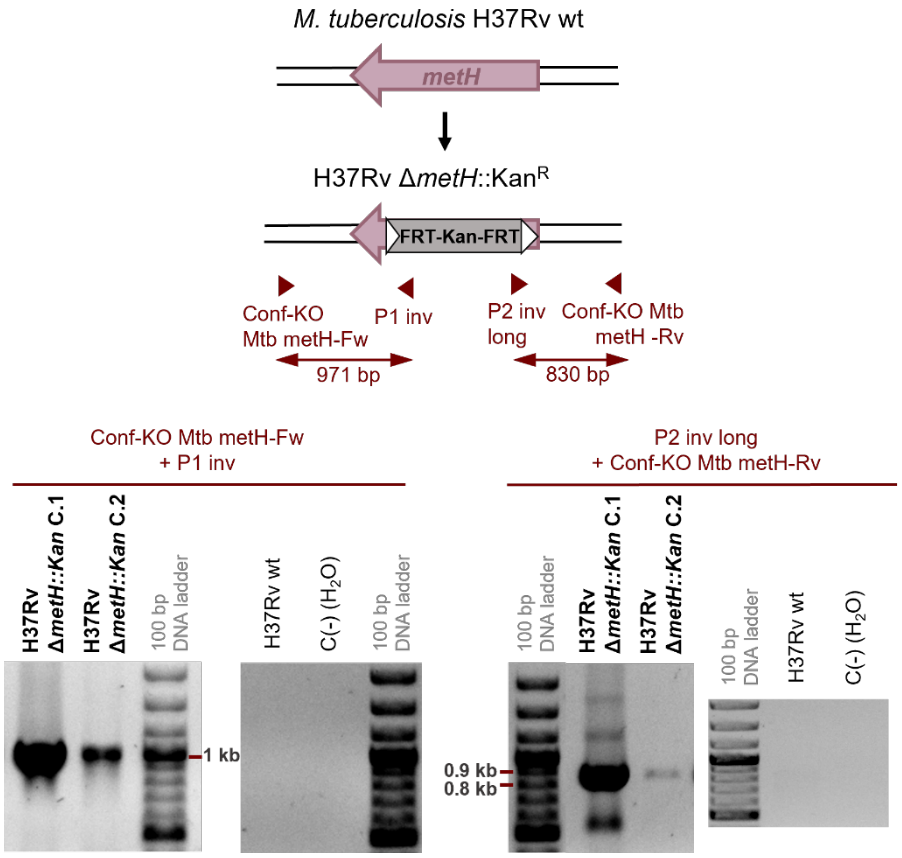

D. Construction of a *M. tuberculosis* Δ*metH* complemented strain with a B12-independent *metE* gene (Pr$_{Ag85A}$-*metE*)

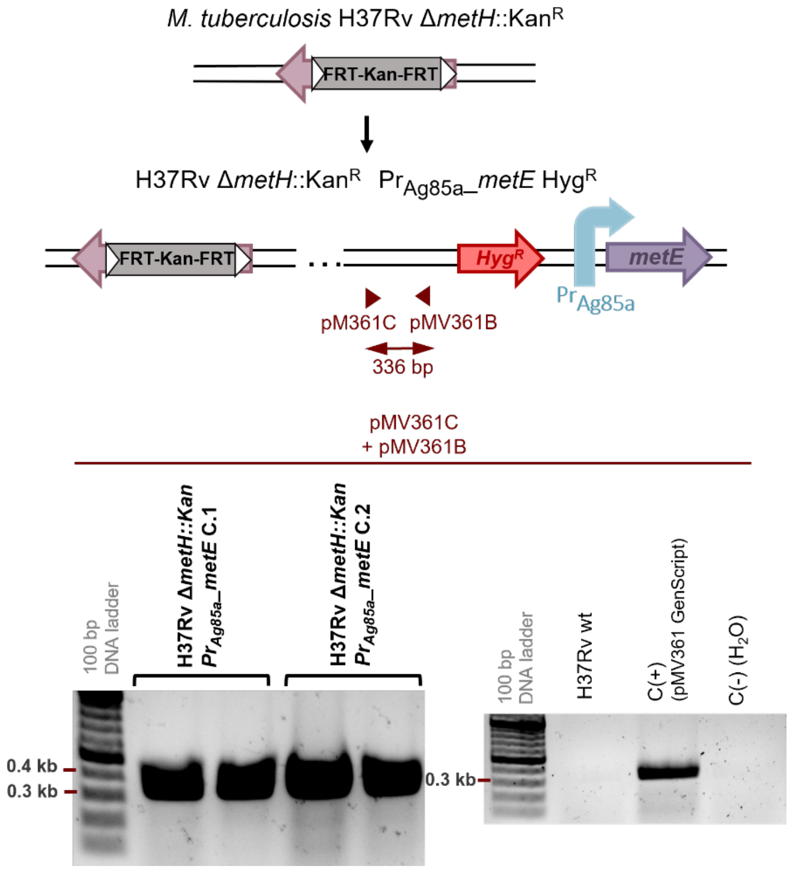

E. Construction of a *M. canettii* Δ*metE* mutant

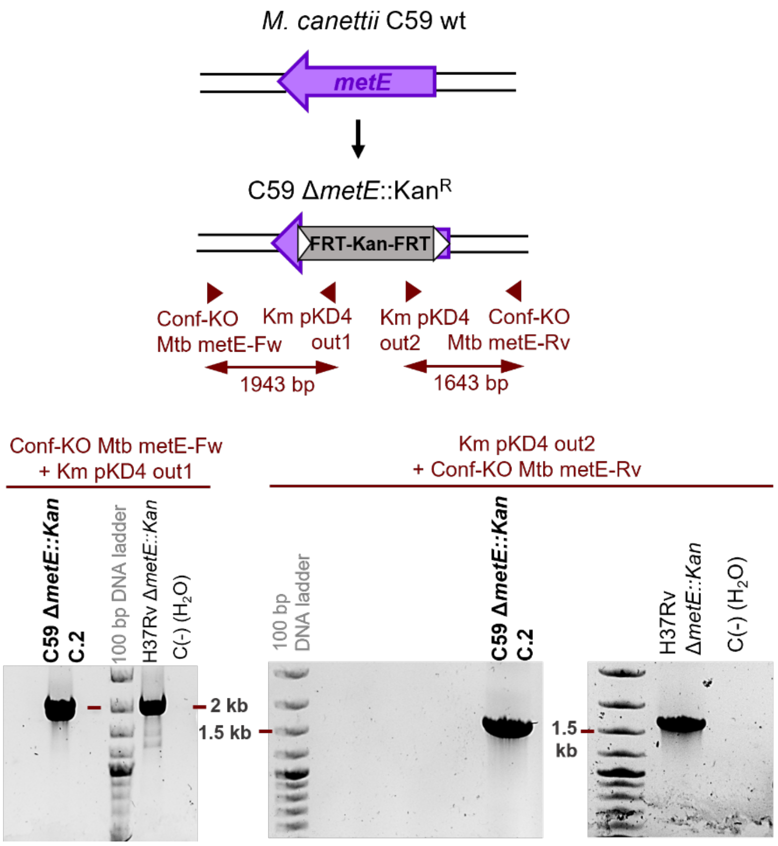

F. Construction of a *M. canettii* Δ*metH* mutant

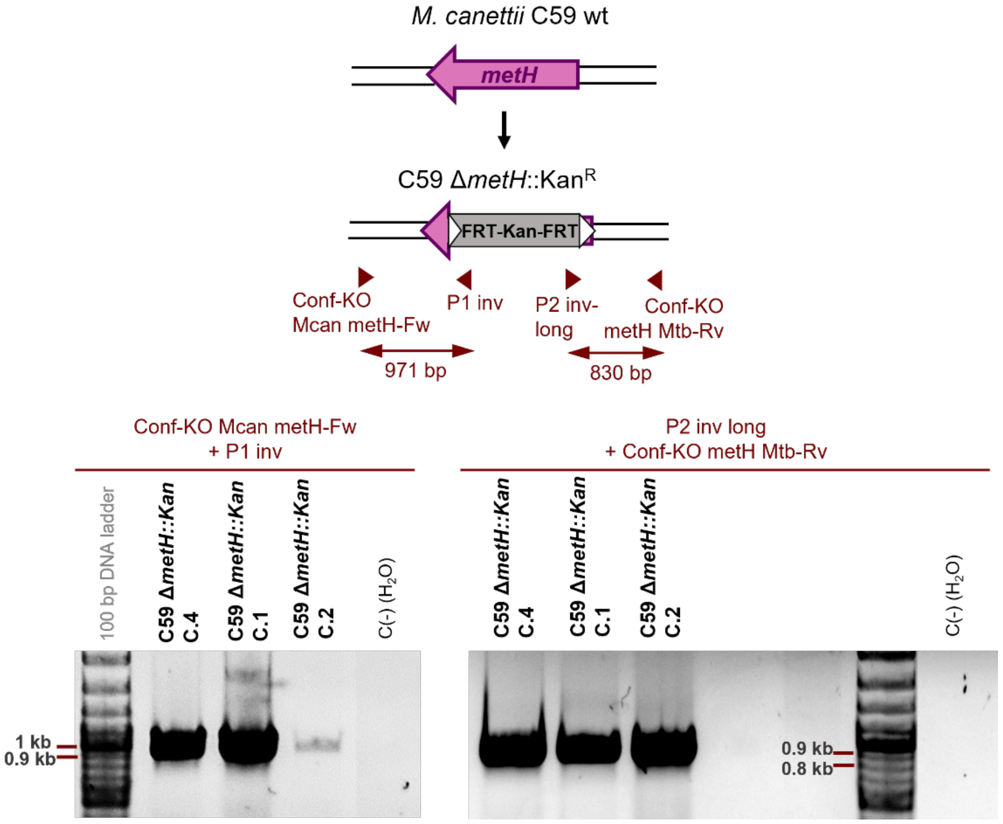

G. Construction of a *M. smegmatis* Δ*cobLMK* mutant

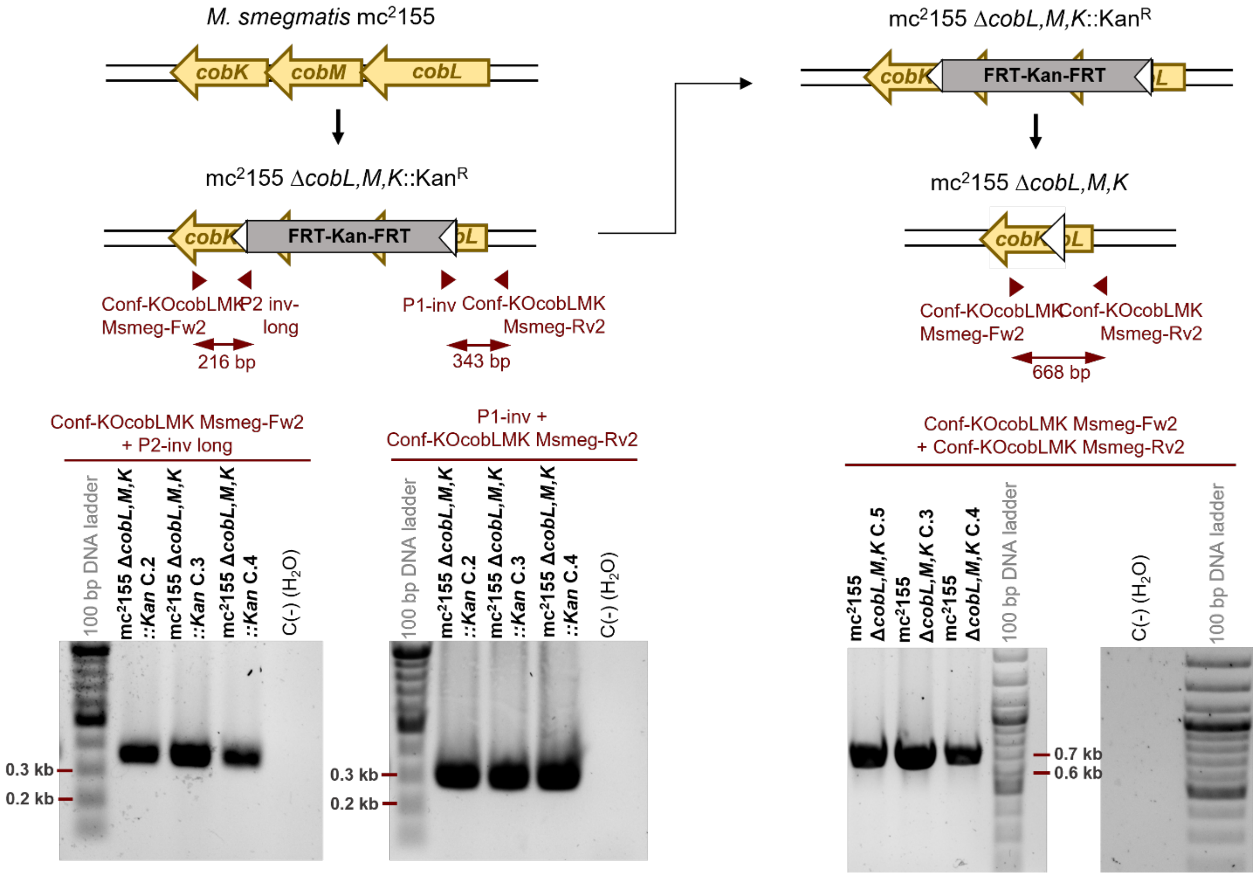

H. Construction of a *M. smegmatis* Δ*cobLMK* Δ*metE* mutant

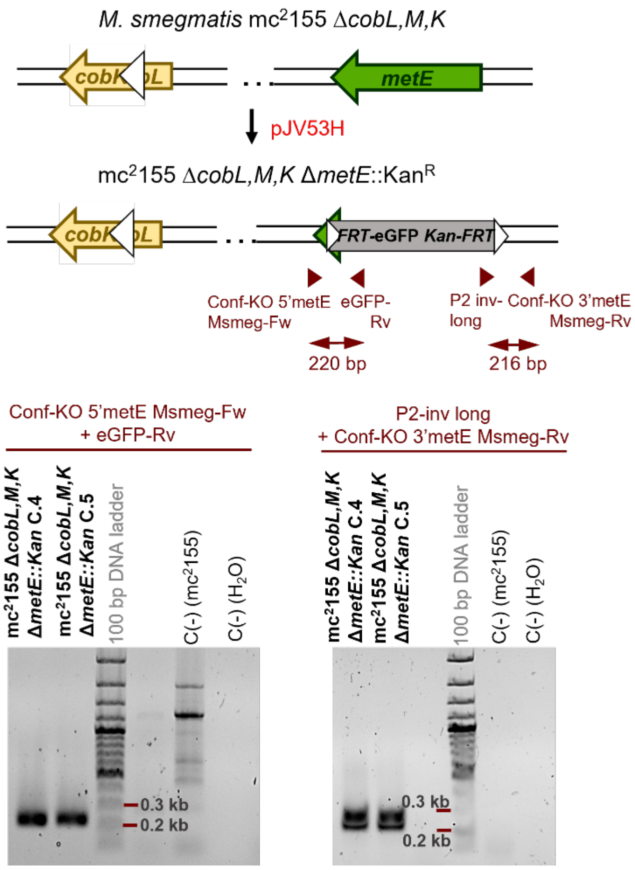

Supplementary Note 1

Genetic products used for construction of mutant and recombinant strains

AES for the construction of ***M. canettii*** **C59 Δ*cobMK*::Kan**[R]

Primers used for PCR amplification:

- Conf KO BAC cobMK Mcan-FW
- Conf KO BAC cobMKMcan-RV

Sequence 5' -> 3':

gcacgacgggcagatcaccaaacacccgatccgcgtgctgaccctggctgcgctggcgccaaggcccgggcagcggttatgggacgtcggcg
cgggctcaggcgccatcgcggtccagtggtgtcggagctggccgggctgcaccgcggtggcgttcgagcgcgacgaacggcgccgccgcaac
attgggttcaatgccgcggccttcggggtgagcgtcgacgtgcgcggcgacgcgcccgatgcgttcgacgacgccgcacggccgtcggtgatt
tttcttggcggtggtgtaacccagccaggcctgcttgaggcctgcctggacagcctgcccgcaggcgggaacttggtcgccaacgctgtcaccg
tcgaatcggaagccgctctggcgcatgcatattcgcgcctcggtggcgagctacgacgattccagcactatctcggcgaaccgctgggcggctt
caccggttggcgcccacagctgccggtcacccagtggtcggtgaccaagcgatgacggtctatttcatcggagcgggccccggcgccgccgac
ctaatcaccgtccgcggccaacggctcctgcaacgatgcccggtgtgcctgtatgcgggttcgatcatgcccgacgacctgttggcgcagtgtc
cgcccggcgcgacgattgtcgacaccggtccgctgaccctcgaacaaatcgtgcgcaaacttgccgacgccgacgccgacggccgcgacgtt
gcccggctgcattccggcgacccgtcgctgtacagcgcgctggccgaacagtgccgcgaactcgacgcgctgggcatcggctacgaaatcgtg
ccgggcgtaccggcttttgccgcagccgcggcggcgctaaagcgcgaactcaccgtgccgggcgtggcgcagacggtgacgctcacccgggt
ggcgacgctgtccacacccataccgcccggtgaggagtgtaggctggagctgcttcgaagttcctatactttctagagaataggaacttcggaa
taggaacttcaagatcccctcacgctgccgcaagcactcagggcgcaagggctgctaaaggaagcggaacacgtagaaagccagtccgcag
aaacggtgctgaccccggatgaatgtcagctactgggctatctggacaagggaaaacgcaagcgcaaagagaaagcaggtagcttgcagtg
ggcttacatggcgatagctagactgggcggttttatggacagcaagcgaaccggaattgccagctggggcgccctctggtaaggttgggaagc
cctgcaaagtaaactggatggctttcttgccgccaaggatctgatggcgcaggggatcaagatctgatcaagagacaggatgaggatcgtttc
gcatgattgaacaagatggattgcacgcaggttctccggccgcttgggtggagaggctattcggctatgactgggcacaacagacaatcggct
gctctgatgccgccgtgttccggctgtcagcgcaggggcgcccggttctttttgtcaagaccgacctgtccggtgccctgaatgaactgcaggac
gaggcagcgcggctatcgtggctggccacgacgggcgttccttgcgcagctgtgctcgacgttgtcactgaagcgggaagggactggctgcta
ttgggcgaagtgccggggcaggatctcctgtcatctcaccttgctcctgccgagaaagtatccatcatggctgatgcaatgcggcggctgcata
cgcttgatccggctacctgcccattcgaccaccaagcgaaacatcgcatcgagcgagcacgtactcggatggaagccggtcttgtcgatcagg
atgatctggacgaagagcatcaggggctcgcgccagccgaactgttcgccaggctcaaggcgcgcatgcccgacggcgaggatctcgtcgtg
acccatggcgatgcctgcttgccgaatatcatggtggaaaatggccgcttttctggattcatcgactgtggccggctgggtgtggcggaccgcta
tcaggacatagcgttggctacccgtgatattgctgaagagcttggcggcgaatgggctgaccgcttcctcgtgctttacggtatcgccgctcccg
attcgcagcgcatcgccttctatcgccttcttgacgagttcttctgagcgggactctggggttcgaaatgaccgaccaagcgacgcccaacctgc
catcacgagatttcgattccaccgccgccttctatgaaaggttgggcttcggaatcgttttccgggacgccggctggatgatcctccagcgcggg
gatctcatgctggagttcttcgcccaccccagcttcaaaagcgctctgaagttcctatactttctagagaataggaacttcggaataggaactaa
ggaggatattcatatgcgcgcaggtgtgcggtgagctcgggctcccgtacctggtactggctcgcccgccgtgggatcccggtaccgccatcat
cgcggtatcggacatcgaggctgcagacgttgttgctgaacaaggttattcgcgagtgttcctgaccaccggacgctcgggtattgcggccttc
gccaacagcgacgcgtggtttttgatccgcgtggttaccgcgcccgatggcaccgccctgccgcggcggcacaaactggtgctatctcgcgggc
catatggttaccacgatgagttcgcactgctgcgtgagcagcgcatcgacgcattggtcaccaagaacagcggtggcaagatgacccgagcg
aagctggatgccgctgctgcgctgggtatttcggtggtcatgatagcgcgcccgctgctgccggctggggtagcggcggtcgattctgtccacc
gggccgccatgtgggtggccggtctgcctagccggtgaggtcgtcgggctccgcgtcggcaagcagcgcatcgcgcgctcgagcgacacggg
atcggatggtgcccaccgggcagccgcacaccgccgcggcgtccgcataggacagcccgagcagctgggtcagcagcagcgcttcccgttgg
tcggtggttaggtcggcgatcatcgtggttacctcgacgaggtcttcgaatccgcgggcatggcggtcgccgtctatgagatgttcgggacgcg
cgccgcgggtggtgcggggccgggatcggacgtggcggatgtgatcggcgacgacatggcgcgcgatggccagcaaccaagttcgggcgct
ggagcgtgcggaaaaccgcgggatggcgccgatcgctcgtaggaatgtctcttgggtgagatcgtccgcactgcccacgtcggacagatagg
cgacgaaccgccacacgtcttgctgggtggctttgataaacgcctcaagggcccgcccgttccccttggccgccgacaaggcgagtgcggtaa

AES for the construction of ***M. tuberculosis*** **H37Rv ΔRv2124c(*metH*)::Kan^{R}**
and ***M. canettii*** **C59 ΔRv2124c(*metH*)::Kan^{R}**

Primers used for PCR amplification:

- Conf-KO BAC metH-FW
- Conf-KO BAC metH-RV

Sequence 5' -> 3':

ttcggtgggtgcgacacatagtgcggcaccgccgcccaaaacgtcaccgccgggatgccggcccccacacaggcagattggaagacg
ccggcgatgccggtggggccctcgtagcgtgtttcttgaaggccgaactgccgcgccgacgccgcggagtaggccgcgcccgagaccg
gcaccggccgggtgtgtggggtgtcggccagcagcgcccccaggatcaccacggtgtccacgttgagtttgtcgatgaccgccagcaac
tcgtcgcaaaacgtgcgccagcgcatattcggctccaccccgcacatcaacaccacgtcgcggtcgctgccgggtgggcggcagtgcg
agatccgcatggccggccactgcagttcccgggtaaccccatcgacttggcggatgaccggccgattgacctggtagtcgtagtaggcc
tcgtcatcgatctcgacaatcggcagtgcttgccagctggccgccaggtgtgccacggcatcgccggccgcgtcgccggcgtcgttccag
ccctcgaacgcagccacgacgacggtgttgtgcagttcgggcagcggtgcgttgccctccgatggggtcacctggtcagcctacggcgt
ccacccgggcgacgatgcgaaccgtgcacgtcgtctgaggcggcccggctagtagcgatggccacgctttgtcgcgctgaggtgggca
cgttgtgtgcctgcctcagctgagactcttgtggcagctgtgaccgatgcgacgtcgcggtacctggcagagcgctgtcaacgactgagg
aaatttcataggccgactatccttgccatgtgtaggctggagctgcttcgaagttcctatactttctagagaataggaacttcggaatagg
aacttcaagatcccctcacgctgccgcaagcactcagggcgcaagggctgctaaaggaagcggaacacgtagaaagccagtccgcag
aaacggtgctgaccccggatgaatgtcagctactgggctatctggacaagggaaaacgcaagcgcaaagagaaagcaggtagcttgc
agtgggcttacatggcgatagctagactgggcggttttatggacagcaagcgaaccggaattgccagctggggcgccctctggtaaggt
tgggaagccctgcaaagtaaactggatggctttcttgccgccaaggatctgatggcgcaggggatcaagatctgatcaagagacagga
tgaggatcgtttcgcatgattgaacaagatggattgcacgcaggttctccggccgcttgggtggagaggctattcggctatgactgggca
caacagacaatcggctgctctgatgccgccgtgttccggctgtcagcgcaggggcgcccggttctttttgtcaagaccgacctgtccggt
gccctgaatgaactgcaggacgaggcagcgcggctatcgtggctggccacgacgggcgttccttgcgcagctgtgctcgacgttgtcac
tgaagcgggaagggactggctgctattgggcgaagtgccggggcaggatctcctgtcatctcaccttgctcctgccgagaaagtatcca
tcatggctgatgcaatgcggcggctgcatacgcttgatccggctacctgcccattcgaccaccaagcgaaacatcgcatcgagcgagca
cgtactcggatggaagccggtcttgtcgatcaggatgatctggacgaagagcatcaggggctcgcgccagccgaactgttcgccaggct
caaggcgcgcatgcccgacggcgaggatctcgtcgtgacccatggcgatgcctgcttgccgaatatcatggtggaaaatggccgctttt
ctggattcatcgactgtggccggctgggtgtggcggaccgctatcaggacatagcgttggctacccgtgatattgctgaagagcttggcg
gcgaatgggctgaccgcttcctcgtgctttacggtatcgccgctcccgattcgcagcgcatcgccttctatcgccttcttgacgagttcttct
gagcgggactctggggttcgaaatgaccgaccaagcgacgcccaacctgccatcacgagatttcgattccaccgccgccttctatgaaa
ggttgggcttcggaatcgttttccgggacgccggctggatgatcctccagcgcggggatctcatgctggagttcttcgcccaccccagctt
caaaagcgctctgaagttcctatactttctagagaataggaacttcggaataggaactaaggaggatattcatatgtctgacccgtccca
cgtgtgcggaagcatcggctgcgacaggacgtcggcgaatccgccgcctaggtgagtaagcccggtcgctgatccgggcgccgattttg
ggatcgtgccggcgaaccccagcgcatccaatccacggttgccggcggcgacgtctgccatctccggtgtgtttggcatggtcaggcgg
ccgtcgtcggccagaaactcgaagcggtgttgccggagacgtcgccgcagtcttgttgctttgctggcgctgctctgagcttggtcgccgg
gcagtttggcggcaggtgcggcggcatcggtggtaacaccggcgagtgctgtcgtcttggcgatcgggttgaacccttccccgtcggga
ccaccgaccgcgtaaaggccttgggcggcactggatccgggagtcgacggtgccgtcggtgccgcggacgtggtcggagcgggtgcgg
gcgtggtaaccagggcagccgggttggtacccaccagggtgactccggtggccgcaggccagctttgccggctgggaccatcggagtg
cacacgttcgaccattggtccggccggccccgataccgcaccgagcccggcaagcccggtcagcccggcaggcaacaggaccggact
cgtcagcggggcgaacggtccgaaatagatcagtgggtccagcaccaggtaggcgagaaacgtgaagactggaccggacacgaacg
agatgaaccagtccaacacctgggcgatga

AES for the construction of ***M. tuberculosis*** **H37Rv ΔRv1133c(*metE*)::Kan^{R}**
and ***M. canettii*** **C59 ΔRv1133c(*metE*)::Kan^{R}**

Primers used for PCR amplification:

- PCR-Frag KO metE Mtb-FW
- PCR-Frag KO metE Mtb-RV

Sequence 5’ -> 3’:

gcgaactccggcttccagaacaccaacaccggcgccttcttcatcggtcactcgggtctgctcaattccggcggcggccagcacgtgggcatcagcaa
ctcgggaaccggctttaacacgggcctgttcaacaccggcttcaacaacaccggcatcggaaactcggccaccaacgccgccttcaccaccacctccg
gggtggccaactccggcgacaacagctcaggcgggttcaacgccggtaatgaccagtccgggttcttcgacggttagcccccggattccagcagcctg
cgccgcgcgagatccttcgcgttggcccgcctgaccgccaccgtcatggcgggtgcggcgattcccgcgaagcgggaaggctcaccacggttttacag
cgtcatcctcgtcgtaccgttcgggccgtcctcccacaggtagaccgtctccatcgggaatggcccttgctcgtccaggtcggccgcctcgtacggaccg
tcgccggtgacgacgtcgccgccgcgggcgccggttacccggaccgtcgtggcggtggccgtcgggtgcagcacggcgccgccacggatcgcggcgg
cgtgctcctgcccgaacttctggtaggcggccatgtcagcggcctgctcgggggccgaccagtcgacgtctctggtgcaggtgagtgcggcgtattgcg
gcctgttgacccatcatggtccgggcctatcccgaatctcacccacatgacgatcgcggcgggcccgatgaggacatccggcgccgagcgattttgcc
gccggccgggccatgccggccgatgtcaccggagtagccttgaccacgcagctggtctgctggcgtccgaaagggcgtcggcatcgagcggggcaac
gatgcttcgcgagagggaacctggtgagaatccgggactgtcccgcagcggtatgcaggaacgaccgccgtcttggaagtagacaagcactggtctc
aacgactgggaagcgacggccagtaggagcacccaccgggtgcgagcctgcgagtccgaagacctgccagccgtgccggacgcgccgcgcccggc
ggctcatcgcctcgtggaatgggcgtttggccgtgcctgttgccgggtgcatcggtgtgcacctcaatcggatcggctgcgcgtccgcgggcggtgaac
cacccgtcgatcgaaggacagcacctcgaggtgtaggctggagctgcttcgaagttcctatactttctagagaataggaacttcggaataggaacttc
aagatcccctcacgctgccgcaagcactcagggcgcaagggctgctaaaggaagcggaacacgtagaaagccagtccgcagaaacggtgctgacc
ccggatgaatgtcagctactgggctatctggacaagggaaaacgcaagcgcaaagagaaagcaggtagcttgcagtgggcttacatggcgatagct
agactgggcggttttatggacagcaagcgaaccggaattgccagctggggcgccctctggtaaggttgggaagccctgcaaagtaaactggatggct
ttcttgccgccaaggatctgatggcgcaggggatcaagatctgatcaagagacaggatgaggatcgtttcgcatgattgaacaagatggattgcacgc
aggttctccggccgcttgggtggagaggctattcggctatgactgggcacaacagacaatcggctgctctgatgccgccgtgttccggctgtcagcgca
ggggcgcccggttctttttgtcaagaccgacctgtccggtgccctgaatgaactgcaggacgaggcagcgcggctatcgtggctggccacgacgggcg
ttccttgcgcagctgtgctcgacgttgtcactgaagcgggaagggactggctgctattgggcgaagtgccggggcaggatctcctgtcatctcaccttgc
tcctgccgagaaagtatccatcatggctgatgcaatgcggcggctgcatacgcttgatccggctacctgcccattcgaccaccaagcgaaacatcgca
tcgagcgagcacgtactcggatggaagccggtcttgtcgatcaggatgatctggacgaagagcatcaggggctcgcgccagccgaactgttcgccag
gctcaaggcgcgcatgcccgacggcgaggatctcgtcgtgacccatggcgatgcctgcttgccgaatatcatggtggaaaatggccgcttttctggatt
catcgactgtggccggctgggtgtggcggaccgctatcaggacatagcgttggctacccgtgatattgctgaagagcttggcggcgaatgggctgacc
gcttcctcgtgctttacggtatcgccgctcccgattcgcagcgcatcgccttctatcgccttcttgacgagttcttctgagcgggactctggggttcgaaat
gaccgaccaagcgacgcccaacctgccatcacgagatttcgattccaccgccgccttctatgaaaggttgggcttcggaatcgttttccgggacgccgg
ctggatgatcctccagcgcggggatctcatgctggagttcttcgcccaccccagcttcaaaagcgctctgaagttcctatactttctagagaataggaac
ttcggaataggaactaaggaggatattcatatgctcgagctgacgagcagctagcccggcagcacgtggaccggcacgtcgtcgtcccagggctgcc
ggttcaccatgtcggccacccggacatagccgcgctcgaactccccggtcgccgcgtctaccaaccggtactcctgggtttgccgatggatgggctgcg
cgtcgagcagcaccacccgcacctcaccgggttggaagccgcaccgctgttgcatcgccgcgatcagttgctcgttgtgcaggtggccgtcgccgaag
ttccagccgacggcagtgctgcagacccgttcgccgtcggtgatgacgtagtcgtcttcgtcatggccggccatcgcccgatgcgccagcgtaaatagc
gccctgccatgggagttcatcgcacgaaacgcgtatcccagatacatcgggatctgggctcggtccttgccgtagaagcgctccagctgagcggccgg
catgctggcgatcgcgacgatgccccggttgatcttgtcctccgcggagggcttgatgcaccacagcgtggtatcccagttgccggcgtaatagcgcat
ggcggctagaaacgagatcttgcggggaaacacgttgcccgcaatgacgattcccgcgacaacggcgatcaggatcgccagcggcaccgggtttttc
acgtccgctaacccgaggcaggcgtggccgacgaacagcgacaggacgccgaagatcatgaacacgttccactccagcggcacccccatcgggatg
gccgtcagaatccccaggtgaaagcagaccatgatcgtcgcggccaccaccgtcggccagccgccgtgcgcaacgaacaacaccacgggcacacac
atctcgataacagtgctgacgtgggcgacaatccgcgacaacagcccgggccgcaggtcgccggggaacttcttgaaaaacatccgcttgatgaacc
gcggccgaaacagcgggttgttggacatcatcgtggagatcacaaaagggaagtgccggttgagtttcgatgtcgccgcaccgatccagatcaccag
gaacaccagtttggcggccacgatcatgtctataccgttcaagcggccgaacaaaaacgtcaccgtcagcgtcgcgtagacctcgccgcgggcggcc
aggaagatcaccttgtcgcgcaggcccagcacaccgagaagcagcaggatcagcacgatctgccaggcg

Genetic construct cloned in pMV361H for ***M. tuberculosis*** **H37Rv ΔRv2124c::Kan^R**
PrAg85a_metE **(Hyg^R)** construction

aagaatctgttcccggactgggtgatcgccgacggaccctgggtgaagtggtacccgggtattcccggctacatcgaccagcagtgggt
cacgcaagccgaagcggccctgcaatgccccgcgactcgggcggtgcttaattctgtgcgcgccccgatcaccctgcaccgattcctctc
caacgtgttgcattcctacgagttcacccgttaccgcatcgaccgcgttccgcgctatgaattggtccggtgtgggctcgatgttcccgac
gggcccggcccgccaccgcgggagtgaggggcaatgagcgcgggggcaatactgacagcaagatcacaattgagccggcactagcg
gtcgacacatgcccagacactgcggaaatgccaccttcaggccgtcgcgtcggtcccgaattggccgtgaacgaccgccggataaggg
tttcggcggtgcgcttgatgcgggtggacgcccgaagttgtggttgactacacgagcactgccgggcccagcgcctgcagtctgaccta
attcaggatgcgcccaaacatgcatggatgcgttgagatgaggatgagggaagcaagagtgacccagcctgtacgtcgtcaacccttt
accgcaaccatcaccggctccccgcgcatcggcccgcgccgcgaactcaagcgcgccaccgaaggctactgggccggacgtaccagc
cgatccgagctggaggccgtcgccgccacgttacgccgcgacacctggtcggccctggccgcggccggtctggactcggtgccggtgaa
caccttctcctactacgaccaaatgctcgataccgcggtgctgctcggcgcgctgccgccccgagtgagcccggtttccgacgggctgga
ccgctatttcgccgcggcgcggggcaccgaccagatcgcgccgctggagatgacgaagtggttcgacaccaactaccactacctggta
cccgagatcgggccgtcgaccacgttcacgctgcaccccggcaaggtgctcgccgaactcaaagaggcgttagggcaaggcattcccg
cacgtccggtgatcatcgggccgatcaccttcctgctgctgagcaaggccgtcgacggcgcgggggcgccgatcgaacgcctcgaaga
gttggttccggtctattcggagctgctgtcgctgcttgccgacggcggcgcccagtgggtgcagttcgacgagccggcgctggtgaccga
cctctcccccgacgcgcccgccctggctgaagcggtgtacaccgcgctgtgctcggtgagcaaccggcctgcgatctatgtcgccaccta
cttcggggacccgggcgcggccctaccggcgctggctcgcaccccggtcgaagccatcggcgtcgacctggtggccggtgccgacacct
cggtggccggggtacccgagctggccggcaagacgctggtggccggggtcgtcgacgggcgcaacgtctggcgcaccgacctggagg
cggcgttgggcacgttggcgaccctgctgggttcggcggctaccgtggccgtctcgacgtcgtgctcgacactgcacgtgccgtactcgc
tggaaccggaaaccgacctggatgacgcgttgcggagctggctggcgttcggtgccgaaaaggtgcgcgaagtcgtcgttctcgcgcgt
gccctgcgcgacggacacgacgcggtcgccgacgagatcgcgtcgtcccgcgccgccatcgcgtcccgcaagcgcgacccgcggttac
acaatgggcaaatccgggcgcgcatcgaggcgatcgtcgcgtccggagcccaccgcggcaatgccgcccagcgccgcgccagccaag
acgcgcgactgcacctgccgccgctgccgaccacgacgatcggctcctacccgcagacctcggcgatccgcgttgcgcgtgcggcgctg
cgggccggtgagatcgacgaggccgagtacgtgcgccggatgcggcaagagatcaccgaggtgatcgcgctacaggagcggctcggg
ctcgacgtgctggtgcacggcgaaccggagcgcaacgacatggtgcagtacttcgccgagcaattggcgggtttcttcgctacccagaa
cggctgggtgcagtcctacggcagccgctgtgtgcgtccgccgatcctgtacggcgacgtgtcccggccgcgggcgatgacggtcgagt
ggatcacctacgcgcagtcgctgaccgacaaaccggtgaagggcatgttgaccgggccggtgacgattctggcgtggtcgttcgtgcgt
gacgaccagccgttggccgataccgccaaccaggtggcgctggcgattcgcgacgagaccgtggatttgcagtccgccggcatcgcgg
tcatccaggtcgacgagcctgcgctgcgtgaactgctgccgctgcgtcgcgccgaccaggccgagtacttgcgttgggcggtaggggct
ttccggttggccacctccggcgtctcggacgccacccagatccacacgcatctgtgctactcggagttcggcgaggtgatcggcgcgatc
gccgatctggacgcggacgtcacgtccatcgaggcggcccggtcacacatggaggtgctcgacgacctgaacgcgatcggcttcgcca
acggtgtgggcccgggcgtctatgacattcactcgccacgggtgccctccgctgaggagatggccgactcgttgcgggccgcgttgcgc
gcggtgccggccgagcggctgtgggtcaaccccgactgcggactgaagacccgcaatgtcgacgaggtgaccgcgtcgctgcacaac
atggtcgccgccgcccgggaggtgcgcgcgggctagctgacgagcagctagcccggcagcacgtggaccggcacgtcgtcgtcccag
ggctgccggttcaccatgtcggccacccggacatagccgcgctcgaactccccggtcgccgcgtctaccaaccggtactcctgggtttgc
cgatggatgggctgcgcgtcgagcagcaccacccgcacctcaccgggttggaagccgcaccgctgttgcatcgccgcgatcagttgctc
gttgtgcaggtggccgtcgccgaagttccagccgacggcagtgctgcagacccgttcgccgtcggtgatgacgtagtcgtcttcgtcatg
gccggccatcgcccgatgcgccagcgtaaatagcgccctgccatgggagttcatcgcacgaaacgcgtatcccagatacatcgggatct
gggctcggtccttgccgtagaagcgctccagctgagcggccggcatgctggcgatcgcgacgatgccccggttgatcttgtcctccgcgg
agggcttgatgcaccacagcgtggtatcccagttgccggcgtaatagcgcatggcggctagaaacgagatcttgcggggaaacacgtt
gccc

AES for the construction of ***M. smegmatis*** **mc^{2}155 Δ*cobLMK*::Kan^R**

Primers used for PCR amplification:

- KO cobLMK Msmeg FRT Kan-FW
- KO cobLMK Msmeg FRT Kan-RV

Sequence 5' -> 3':

ctggcgcaggatgccgcgatgagcgctcatgcgaagagccgaggacaccggtgtaggctggagctgcttcgaagttcctatactttcta
gagaataggaacttcggaataggaacttcaagatcccctcacgctgccgcaagcactcagggcgcaagggctgctaaaggaagcgga
acacgtagaaagccagtccgcagaaacggtgctgaccccggatgaatgtcagctactgggctatctggacaagggaaaacgcaagcg
caaagagaaagcaggtagcttgcagtgggcttacatggcgatagctagactgggcggttttatggacagcaagcgaaccggaattgcc
agctggggcgccctctggtaaggttgggaagccctgcaaagtaaactggatggctttcttgccgccaaggatctgatggcgcaggggat
caagatctgatcaagagacaggatgaggatcgtttcgcatgattgaacaagatggattgcacgcaggttctccggccgcttgggtggag
aggctattcggctatgactgggcacaacagacaatcggctgctctgatgccgccgtgttccggctgtcagcgcaggggcgcccggttctt
tttgtcaagaccgacctgtccggtgccctgaatgaactgcaggacgaggcagcgcggctatcgtggctggccacgacgggcgttccttg
cgcagctgtgctcgacgttgtcactgaagcgggaagggactggctgctattgggcgaagtgccggggcaggatctcctgtcatctcacct
tgctcctgccgagaaagtatccatcatggctgatgcaatgcggcggctgcatacgcttgatccggctacctgcccattcgaccaccaagc
gaaacatcgcatcgagcgagcacgtactcggatggaagccggtcttgtcgatcaggatgatctggacgaagagcatcaggggctcgcg
ccagccgaactgttcgccaggctcaaggcgcgcatgcccgacggcgaggatctcgtcgtgacccatggcgatgcctgcttgccgaatat
catggtggaaaatggccgcttttctggattcatcgactgtggccggctgggtgtggcggaccgctatcaggacatagcgttggctacccg
tgatattgctgaagagcttggcggcgaatgggctgaccgcttcctcgtgctttacggtatcgccgctcccgattcgcagcgcatcgccttc
tatcgccttcttgacgagttcttctgagcgggactctggggttcgaaatgaccgaccaagcgacgcccaacctgccatcacgagatttcg
attccaccgccgccttctatgaaaggttgggcttcggaatcgttttccgggacgccggctggatgatcctccagcgcggggatctcatgct
ggagttcttcgcccaccccagcttcaaaagcgctctgaagttcctatactttctagagaataggaacttcggaataggaactaaggagg
atattcatatggtggtggcgcagcgcgggttttcgcgcgtgttcctgaccacggggcgctc

AES for the construction of ***M. smegmatis*** **mc^{2}155 Δ*cobLMK* Δ*metE*::eGFP_Kan^R**

Primers used for PCR amplification:

- PCR-Frag GFP+Kan KO metE Msmeg-FW
- PCR-Frag GFP+Kan KO metE Msmeg-RV

Sequence 5' -> 3':

gaagacctgccagccgtaccggatgcgccgcgtccggtggttcatcgcctcgtggaatgggcattggccgaatcggtagacctgtcgtg
gtcagccgcgttcggttcgacgtctcaaggcggtgagcaagggcgaggagctgttcaccggggtggtgcccatcctggtcgagctggac
ggcgacgtaaacggccacaagttcagcgtgtccggcgagggcgagggcgatgccacctacggcaagctgaccctgaagttcatctgca
ccaccggcaagctgcccgtgccctggcccaccctcgtgaccaccctgacctacggcgtgcagtgcttcagccgctaccccgaccacatg
aagcagcacgacttcttcaagtccgccatgcccgaaggctacgtccaggagcgcaccatcttcttcaaggacgacggcaactacaaga
cccgcgccgaggtgaagttcgagggcgacaccctggtgaaccgcatcgagctgaagggcatcgacttcaaggaggacggcaacatcc
tggggcacaagctggagtacaactacaacagccacaacgtctatatcatggccgacaagcagaagaacggcatcaaggtgaacttca
agatccgccacaacatcgaggacggcagcgtgcagctcgccgaccactaccagcagaacacccccatcggcgacggccccgtgctgct
gcccgacaaccactacctgagcacccagtccgccctgagcaaagaccccaacgagaagcgcgatcacatggtcctgctggagttcgtg
accgccgccgggatcactctcggcatggacgagctgtacaagtaataaatcgatgtcgacgtagttaactagcgtacgatcgactgcca
ggcatcaaataaaacgaaaggctcagtcgaaagactgggcctttcgttttatctcgaggtgtaggctggagctgcttcgaagttcctata
ctttctagagaataggaacttcggaataggaacttcaagatcccctcacgctgccgcaagcactcagggcgcaagggctgctaaagga
agcggaacacgtagaaagccagtccgcagaaacggtgctgaccccggatgaatgtcagctactgggctatctggacaagggaaaacg
caagcgcaaagagaaagcaggtagcttgcagtgggcttacatggcgatagctagactgggcggttttatggacagcaagcgaaccgg
aattgccagctggggcgccctctggtaaggttgggaagccctgcaaagtaaactggatggctttcttgccgccaaggatctgatggcgc
aggggatcaagatctgatcaagagacaggatgaggatcgtttcgcatgattgaacaagatggattgcacgcaggttctccggccgcttg
ggtggagaggctattcggctatgactgggcacaacagacaatcggctgctctgatgccgccgtgttccggctgtcagcgcaggggcgcc
cggttctttttgtcaagaccgacctgtccggtgccctgaatgaactgcaggacgaggcagcgcggctatcgtggctggccacgacgggc
gttccttgcgcagctgtgctcgacgttgtcactgaagcgggaagggactggctgctattgggcgaagtgccggggcaggatctcctgtca
tctcaccttgctcctgccgagaaagtatccatcatggctgatgcaatgcggcggctgcatacgcttgatccggctacctgcccattcgacc
accaagcgaaacatcgcatcgagcgagcacgtactcggatggaagccggtcttgtcgatcaggatgatctggacgaagagcatcagg
ggctcgcgccagccgaactgttcgccaggctcaaggcgcgcatgcccgacggcgaggatctcgtcgtgacccatggcgatgcctgcttg
ccgaatatcatggtggaaaatggccgcttttctggattcatcgactgtggccggctgggtgtggcggaccgctatcaggacatagcgttg
gctacccgtgatattgctgaagagcttggcggcgaatgggctgaccgcttcctcgtgctttacggtatcgccgctcccgattcgcagcgca
tcgccttctatcgccttcttgacgagttcttctgagcgggactctggggttcgaaatgaccgaccaagcgacgcccaacctgccatcacg
agatttcgattccaccgccgccttctatgaaaggttgggcttcggaatcgttttccgggacgccggctggatgatcctccagcgcggggat
ctcatgctggagttcttcgcccaccccagcttcaaaagcgctctgaagttcctatactttctagagaataggaacttcggaataggaacta
aggaggatattcatatgctcgagtccctgcgggtggtggccccggtccgacgccggggccaccacccggagaagtcactcaggggccg
cgctggaacgcgagaccgtcatcgcgatggtctggag

SUPPLEMENTARY TABLES

SUPPLEMENTARY TABLE 1. Bacterial strains used and constructed in this study

Mycobacterial strains used			
**Species & strain designation**	**Description**	**Use in this study**	**Reference**
***M. smegmatis* mc²155**	Reference laboratory strain of *M. smegmatis*	*In vitro* B12 production experiments	1
M. mucogenicum	Strain provided by Hospital Miguel Servet (Zaragoza)	*In vitro* B12 production experiments	This study
M. fortuitum	Strain provided by Hospital Miguel Servet (Zaragoza)	*In vitro* B12 production experiments	This study
M. xenopii	Strain provided by Hospital Miguel Servet (Zaragoza)	*In vitro* B12 production experiments	This study
M. abscesus	Strain provided by Hospital Miguel Servet (Zaragoza)	*In vitro* B12 production experiments	This study
M. avium	Strain provided by Hospital Miguel Servet (Zaragoza)	*In vitro* B12 production experiments	This study
M. gordonae	Strain provided by Hospital Miguel Servet (Zaragoza)	*In vitro* B12 production experiments	This study
***M. canettii* C59 (STB-A) (CIPT 140010059)**	Clinical isolate of *M. canettii*	*In vitro* B12 production and uptake experiments. *In vivo* experiments in mice	2
***M. africanum* HCU2744**	Clinical isolate of *M. africanum* lineage 5 provided by Hospital Clínico Universitario Lozano Blesa (Zaragoza)	*In vitro* B12 production experiments	This study
***M. africanum* HCU2828**	Clinical isolate of *M. africanum* lineage 6 provided by Hospital Clínico Universitario Lozano Blesa (Zaragoza)	*In vitro* B12 production and uptake experiments	This study
***M. bovis* AF2122/97**	Reference laboratory strain of *M. bovis* clade A4	*In vitro* B12 production and uptake experiments	3
***M. bovis* AN5**	Reference laboratory strain of *M. bovis* clade A4	*In vitro* B12 production experiments	4
***M. tuberculosis* GC1237**	Clinical isolate of *M. tuberculosis* lineage 2	*In vitro* B12 production experiments	5
***M. tuberculosis* Mt103**	Clinical isolate of *M. tuberculosis* lineage 4	*In vitro* B12 production experiments	6
***M. tuberculosis* H37Rv**	Reference laboratory strain of *M. tuberculosis* lineage 4	*In vitro* B12 production and uptake experiments. *In vitro* growth characterization experiments. *In vivo* experiments in mice	7
E. coli strains used			
***E. coli* DH10B**	*E. coli* strain carrying a *M. tuberculosis* H37Rv BAC library	BAC-rec for KO construction	8

***E. coli* DH10B BAC Rv412 pKD46**	Clone carrying recombineering plasmid pKD46 and BAC Rv412 containing *cobMK* genes	BAC-rec for KO construction in *M. canettiii*	This study
***E. coli* DH10B BAC Rv73 pKD46**	Clone carrying recombineering plasmid pKD46 and BAC Rv73 containing Rv2124c *(metH)* gene	BAC-rec for KO construction in *M. tuberculosis* & *M. canettii*	This study

Mycobacterial mutant strains constructed in this study

**Species & strain designation**	**Description**	**Use in this study**	**Construction method**
***M. canettii* C59 Δ*cobMK*::Kan^R**	C59 mutant of *cobMK* genes with a FRT-Kan^R-FRT cassette	*In vivo* experiments in mice	BAC-recombineering
***M. tuberculosis* H37Rv Δ*metE*::Kan^R**	H37Rv mutant of *metE* (Rv1133c) gene with a FRT-Kan^R-FRT cassette	*In vitro* growth characterization experiments. *In vivo* experiments in mice	Recombineering of synthetic AES
***M. tuberculosis* H37Rv Δ*metH*::Kan^R**	H37Rv mutant of *metH* gene (Rv2124c) with a FRT-Kan^R-FRT cassette	*In vitro* growth characterization experiments. *In vivo* experiments in mice	BAC- recombineering
***M. tuberculosis* H37Rv Δ*metH*::Kan^R Pr$_{Ag85a}$_*metE* (Hyg^R)**	Complemented copy of the *metE* gene controlled by *fbpA* (Ag85a) promoter in H37Rv Δ*metH*::*Kan^R*	*In vitro* growth characterization experiments. *In vivo* experiments in mice	Integration of pMV361H-Pr$_{Ag85a}$_*metE* plasmid
***M. canettii* C59 Δ*metE*::Kan^R**	C59 mutant of *metE* gene with a FRT-Kan^R-FRT cassette	*In vitro* growth characterization experiments.	Recombineering of synthetic AES
***M. canettii* C59 Δ*metH*::Kan^R**	C59 mutant of *metH* gene with a FRT-Kan^R-FRT cassette	*In vitro* growth characterization experiments	BAC- recombineering
***M. smegmatis* mc^{2}155 Δ*cobLMK* Δ*metE*::eGFP_Kan^R**	mc^{2}155 Δ*cobLMK* mutant of *metE* gene with a FRT-Kan^R-FRT cassette and eGFP gene	*In vitro* growth characterization experiments	Recombineering of PCR-constructed AES

SUPPLEMENTARY TABLE 2. Primers used in this study

qRT-PCR analysis

Primer	Sequence 5' -> 3'	Use in this study	Reference
**qRT-Rv1129c-FW**	ATGTCAACCAGTTGGAGAATGA	qRT-PCR verification of "Core-B12 regulon" expression	This study
**qRT-Rv1129c-RV**	CGGAATCCGAGGAGAAATACTG	qRT-PCR verification of "Core-B12 regulon" expression	This study
**qRT-prpD-FW**	CTGCGTATGGTGCGGATTAT	qRT-PCR verification of "Core-B12 regulon" expression	This study
**qRT-prpD-RV**	GCGATCTTGTAGGCCATGT	qRT-PCR verification of "Core-B12 regulon" expression	This study
**qRT-PPE2-FW**	ATCCCGCAACTATCGCTTT	qRT-PCR verification of "Core-B12 regulon" expression	This study
**qRT-PPE2-RV**	GCGATGGTATCGGTGATGAT	qRT-PCR verification of "Core-B12 regulon" expression	This study
**qRT-cobQ1-FW**	GCAGAACATGTCCAACAACTC	qRT-PCR verification of "Core-B12 regulon" expression	This study
**qRT-cobQ1-RV**	AGCAAGACCGGGTTCATC	qRT-PCR verification of "Core-B12 regulon" expression	This study
**qRT-metE-FW**	GTTCGACACCAACTACCACTAC	qRT-PCR verification of "Core-B12 regulon" expression and qRT-PCR of *metE* expression in L-Met synthesis mutants	This study
**qRT-metE-RV**	CTTGCCCTAACGCCTCTTT	qRT-PCR verification of "Core-B12 regulon" expression and qRT-PCR of *metE* expression in L-Met synthesis mutants	This study
**RT-sigA-FW**	CCGATGACGACGAGGAGATC	Normalization of gene expression in qRT-PCR analysis	[9]
**RT-sigA-RV**	CGGAGGCCTTGTCCTTTTC	Normalization of gene expression in qRT-PCR analysis	[9]

Knockouts construction and PCRs of verification

Primer	Sequence 5' -> 3'	Use in this study	Reference
**KO BAC cobMK Mcan P1-Fw**	ACGCTCACCCGGGTGGCGACGCTG TCACCCCATACCGCCCGGTGAGGA GTGTAGGCTGGAGCTGCTTC	AES synthesis PCR for KO of *cobMK* in *E. coli* DH10B BAC Rv412 pKD46	This study
**KO BAC cobMK Mcan P1-Rv**	GGCGAGCCAGTACCAGGTACGGGA GCCCGAGCTCACCGCACACCTGCGC GCATATGAATATCCTCCTTAGT	AES synthesis PCR for KO of *cobMK* in *E. coli* DH10B BAC Rv412 pKD46	This study
**P1-inv**	GAAGCAGCTCCAGCCTACAC	PCR verification of deleted genes, inside Kan^R cassette	[10]
**P2-inv long**	CTTCGGAATAGGAACTAAGGAGG ATATTCATATG	PCR verification of deleted genes, inside Kan^R cassette	[10]
**Conf-KO BAC cobMK Mcan-Fw**	GCACGACGGGCAGATCACCAAA	PCR verification of *cobMK* deletion in *E. coli* and AES amplification PCR for KO of *cobMK* in *M. canettii*	This study
**Conf-KO BAC cobMK Mcan-Rv**	TTACCGCACTCGCCTTGTCG	PCR verification of *cobMK* deletion in *E. coli* and AES amplification PCR for KO of *cobMK* in *M. canettii*	This study
**Confirm-KO cobMK C59-Fw**	GTTCAGCGTGCTCGAACAGCTT	PCR verification flanking *cobMK* deletion in *M. canettii*	This study
**Confirm-KO cobMK C59-Rv**	TCTACGACGACCGGTCGTAGAC	PCR verification flanking *cobMK* deletion in *M. canettii*	This study
**KO BAC Rv2124c FRT Kan-FW**	GAGCGCTGTCAACGACTGAGGAA ATTTCATAGGCCGACTATCCTTGC CATGTGTAGGCTGGAGCTGCTTC	AES synthesis PCR for KO of Rv2124c (*metH*) in *E. coli* DH10B BAC Rv73 pKD46	This study

**KO BAC Rv2124c FRT Kan-RV**	GCCGACGTCCTGTCGCAGCCGATG CTCCGCACACGTGGGACGGGTCAG A CATATGAATATCCTCCTTAGT	AES synthesis PCR for KO of Rv2124c (*metH*) in *E. coli* DH10B BAC Rv73 pKD46	This study
**Conf-KO BAC metH-FW**	TTCGGTGGGTGCGACACATAGT	PCR verification of *metH* deletion in *E. coli* and AES amplification PCR for KO of *metH* in *M. tuberculosis*	This study
**Conf-KO BAC metH-RV**	TCATCGCCCAGGTGTTGGACTG	PCR verification of *metH* deletion in *E. coli* and AES amplification PCR for KO of *metH* in *M. tuberculosis*	This study
**Conf-KO Mtb metH-FW**	TTCGGCGATCGTCTCGGTGATC	PCR verification flanking *metH* deletion in *M. tuberculosis*	This study
**Conf-KO Mtb metH-RV**	TGCGCTATCTGGCTGTTGAGCT	PCR verification flanking *metH* deletion in *M. tuberculosis* and in *M. canettii*	This study
**PCR-Frag KO metE Mtb-FW**	GCGAACTCCGGCTTCCAGAACA	AES amplification PCR for KO of *metE* in *M. tuberculosis*	This study
**PCR-Frag KO metE Mtb-RV**	CGCCTGGCAGATCGTGCTG	AES amplification PCR for KO of *metE* in *M. tuberculosis*	This study
**Conf-KO Mtb metE-FW**	ACTCCGGAGACCTGAACACCG	PCR verification flanking *metE* deletion in *M. tuberculosis* and in *M. canettii*	This study
**Conf-KO Mtb metE-RV**	TTGCCCTCTACGCACTGCTG	PCR verification flanking *metE* deletion in *M. tuberculosis* and in *M. canettii*	This study
**Km pKD4 out1**	CCACGATAGCCGCGCTGCCTCG	PCR verification of deleted genes, inside Kan^R cassette	[11]
**Km pKD4 out2**	GGGCTGACCGCTTCCTCGTGCT	PCR verification of deleted genes, inside Kan^R cassette	[11]
**pMV361C**	GATCCGGAGGAATCACTTC	PCR verification of integration of pMV361H-Pr_{Ag85a}_metE plasmid in *M. tuberculosis* Δ*metH*:: Kan^R	[12]
**pMV361B**	CCTCGAGCAAGACGTTTCC	PCR verification of integration of pMV361H-Pr_{Ag85a}_metE plasmid in *M. tuberculosis* Δ*metH*:: Kan^R	[12]
**Conf-KO Mcan metH-Fw**	ACCTCGACGTCGAGCACG	PCR verification flanking *metH* deletion in *M. canettii*	This study
**KO cobLMK Msmeg FRT Kan-FW**	CTGGCGCAGGATGCCGCGATGAGC GCTCATGCGAAGAGCCGAGGACAC CGGTGTAGGCTGGAGCTGCTTC	AES synthesis PCR for KO of *cobLMK* in *M. smegmatis*	This study
**KO cobLMK Msmeg FRT Kan-RV**	GAGCGCCCCGTGGTCAGGAACACG CGCGAAAACCCGCGCTGCGCCACC ACCATATGAATATCCTCCTTAGT	AES synthesis PCR for KO of *cobLMK* in *M. smegmatis*	This study
**Conf-KO cobLMK Msmeg-FW2**	TCCAGTTTGGGCTGTGTCATGTC	PCR verification flanking *cobLMK* deletion in *M. smegmatis*	This study
**Conf-KO cobLMK Msmeg-RV2**	TTATGTCTACGGTTCGGCCAAGG	PCR verification flanking *cobLMK* deletion in *M. smegmatis*	This study
**PCR-Frag GFP+Kan KO metE Msmeg-FW**	GAAGACCTGCCAGCCGTACC	AES synthesis PCR for KO of *metE* in *M. smegmatis*	This study
**PCR-Frag GFP+Kan KO metE Msmeg-RV**	CTCCAGACCATCGCGATGACG	AES synthesis PCR for KO of *metE* in *M. smegmatis*	This study
**Conf-KO 5'metE Msmeg-FW**	AGTAGGTGTGCGTCAGCGCA	PCR verification flanking *metE* deletion in *M. smegmatis*	This study

**Conf-KO 3'metE Msmeg-RV**	TCGGGCTTGTCAACTTGTCAGG	PCR verification flanking *metE* deletion in *M. smegmatis*	This study
**eGFP-RV**	CGTCGCCGTCCAGCTCGACCAG	PCR verification flanking *metE* deletion in *M. smegmatis,* inside eGFP gene	[12]

SUPPLEMENTARY TABLE 3. Plasmids used in this study

Plasmid	Description	Reference
**pKD46**	Thermosensitive and replicative plasmid for *E. coli* containing the λ–red recombinase system from lambda phage inducible by arabinose, and an Amp-resistance marker	[13]
**pKD4**	Replicative plasmid for *E. coli* containing the FRT-Kan-FRT resistance marker	[13]
**pJV53H**	Replicative plasmid for *E. coli* and *Mycobacterium* containing Che9c-gp60-61 recombinase system inducible by acetamide and a Hyg-resistance marker	[14]
**pRES-FLP-Mtb**	Replicative plasmid for *E. coli* and *Mycobacterium* containing the *flp* recombinase from *Saccharomyces cerevisiae* with codon usage adapted to *Mycobacterium* and a Hyg-resistance marker	[15] [10]
**pMV361H-PrAg85a_metE**	Integrative mycobacterial vector with attP site and *int* gene from mycobacteriophage L5, an origin of replication for *E. coli,* a Kan-resistance marker and a genetic construction cloned in the *Nde*I site containing the CDS of H37Rv *metE* gene controlled by the H37Rv *fbpA* promoter	This study

SUPPLEMENTARY TABLES REFERENCES

1 Snapper, S. B., Melton, R. E., Mustafa, S., Kieser, T. & Jacobs, W. R., Jr. Isolation and characterization of efficient plasmid transformation mutants of Mycobacterium smegmatis. *Mol Microbiol* **4**, 1911-1919, doi:10.1111/j.1365-2958.1990.tb02040.x (1990).

2 Supply, P. *et al.* Genomic analysis of smooth tubercle bacilli provides insights into ancestry and pathoadaptation of Mycobacterium tuberculosis. *Nat Genet* **45**, 172-179, doi:10.1038/ng.2517 (2013).

3 Garnier, T. *et al.* The complete genome sequence of Mycobacterium bovis. *Proc Natl Acad Sci U S A* **100**, 7877-7882, doi:10.1073/pnas.1130426100 (2003).

4 Keating, L. A. *et al.* The pyruvate requirement of some members of the Mycobacterium tuberculosis complex is due to an inactive pyruvate kinase: implications for in vivo growth. *Mol Microbiol* **56**, 163-174, doi:10.1111/j.1365-2958.2005.04524.x (2005).

5 Caminero, J. A. *et al.* Epidemiological evidence of the spread of a Mycobacterium tuberculosis strain of the Beijing genotype on Gran Canaria Island. *Am J Respir Crit Care Med* **164**, 1165-1170, doi:10.1164/ajrccm.164.7.2101031 (2001).

6 Jackson, M. *et al.* Inactivation of the antigen 85C gene profoundly affects the mycolate content and alters the permeability of the Mycobacterium tuberculosis cell envelope. *Mol Microbiol* **31**, 1573-1587, doi:10.1046/j.1365-2958.1999.01310.x (1999).

7 Cole, S. T. *et al.* Deciphering the biology of Mycobacterium tuberculosis from the complete genome sequence. *Nature* **393**, 537-544, doi:10.1038/31159 (1998).

8 Brosch, R. *et al.* Use of a Mycobacterium tuberculosis H37Rv bacterial artificial chromosome library for genome mapping, sequencing, and comparative genomics. *Infect Immun* **66**, 2221-2229, doi:10.1128/iai.66.5.2221-2229.1998 (1998).

9 Gonzalo-Asensio, J. *et al.* PhoP: a missing piece in the intricate puzzle of Mycobacterium tuberculosis virulence. *PLoS One* **3**, e3496, doi:10.1371/journal.pone.0003496 (2008).

10 Perez, I. *et al.* Live attenuated TB vaccines representing the three modern Mycobacterium tuberculosis lineages reveal that the Euro-American genetic background confers optimal vaccine potential. *EBioMedicine* **55**, 102761, doi:10.1016/j.ebiom.2020.102761 (2020).

11 Broset, E. *et al.* MTBVAC-Based TB-HIV Vaccine Is Safe, Elicits HIV-T Cell Responses, and Protects against Mycobacterium tuberculosis in Mice. *Mol Ther Methods Clin Dev* **13**, 253-264, doi:10.1016/j.omtm.2019.01.014 (2019).

12 Broset, E. *et al.* Engineering a new vaccine platform for heterologous antigen delivery in live-attenuated Mycobacterium tuberculosis. *Comput Struct Biotechnol J* **19**, 4273-4283, doi:10.1016/j.csbj.2021.07.035 (2021).

13 Datsenko, K. A. & Wanner, B. L. One-step inactivation of chromosomal genes in Escherichia coli K-12 using PCR products. *Proc Natl Acad Sci U S A* **97**, 6640-6645, doi:10.1073/pnas.120163297 (2000).

14 van Kessel, J. C. & Hatfull, G. F. Recombineering in Mycobacterium tuberculosis. *Nat Methods* **4**, 147-152, doi:10.1038/nmeth996 (2007).

15 Song, H. & Niederweis, M. Functional expression of the Flp recombinase in Mycobacterium bovis BCG. *Gene* **399**, 112-119, doi:10.1016/j.gene.2007.05.005 (2007).
