# Supplementary material for: Dependency on host vitamin B12 has shaped Mycobacterium tuberculosis Complex evolution
Source: Nat Commun. 2024 Mar 9;15:2161. doi: 10.1038/s41467-024-46449-8 (PMC10924821; doi:10.1038/s41467-024-46449-8)
Supplement: Supplementary file 3 — Description of Additional Supplementary Files [file 41467_2024_46449_MOESM3_ESM.pdf]

Description of Additional Supplementary Files

File Name: Supplementary Data 1
Description: Differentially expressed genes in the presence of vitamin B12 in the *M. bovis* AF2122/97 strain

File Name: Supplementary Data 2
Description: Differentially expressed genes in the presence of vitamin B12 in the *M. tuberculosis* Beijing GC1237 strain

File Name: Supplementary Data 3
Description: Differentially expressed genes in the presence of vitamin B12 in the *M. tuberculosis* H37Rv strain
