# Supplementary material for: Dependency on host vitamin B12 has shaped Mycobacterium tuberculosis Complex evolution
Source: Nat Commun. 2024 Mar 9;15:2161. doi: 10.1038/s41467-024-46449-8 (PMC10924821; doi:10.1038/s41467-024-46449-8)
Supplement: Supplementary file 7 — Reporting Summary [file 41467_2024_46449_MOESM7_ESM.pdf]

nature portfolio

Corresponding author(s): JESÚS GONZALO-ASENSIO

Last updated by author(s): Feb 21, 2024

Reporting Summary

Nature Portfolio wishes to improve the reproducibility of the work that we publish. This form provides structure for consistency and transparency in reporting. For further information on Nature Portfolio policies, see our Editorial Policies and the Editorial Policy Checklist.

Statistics

For all statistical analyses, confirm that the following items are present in the figure legend, table legend, main text, or Methods section.

n/a	Confirmed	
☐	☒	The exact sample size (*n*) for each experimental group/condition, given as a discrete number and unit of measurement
☒	☐	A statement on whether measurements were taken from distinct samples or whether the same sample was measured repeatedly
☐	☒	The statistical test(s) used AND whether they are one- or two-sided<br>*Only common tests should be described solely by name; describe more complex techniques in the Methods section.*
☒	☐	A description of all covariates tested
☒	☐	A description of any assumptions or corrections, such as tests of normality and adjustment for multiple comparisons
☐	☒	A full description of the statistical parameters including central tendency (e.g. means) or other basic estimates (e.g. regression coefficient) AND variation (e.g. standard deviation) or associated estimates of uncertainty (e.g. confidence intervals)
☐	☒	For null hypothesis testing, the test statistic (e.g. *F*, *t*, *r*) with confidence intervals, effect sizes, degrees of freedom and *P* value noted<br>*Give P values as exact values whenever suitable.*
☒	☐	For Bayesian analysis, information on the choice of priors and Markov chain Monte Carlo settings
☒	☐	For hierarchical and complex designs, identification of the appropriate level for tests and full reporting of outcomes
☒	☐	Estimates of effect sizes (e.g. Cohen's *d*, Pearson's *r*), indicating how they were calculated

Our web collection on statistics for biologists contains articles on many of the points above.

Software and code

Policy information about availability of computer code

Data collection	No software was used for data collection
Data analysis	Statistical analyses were performed using Prism 9.5.1

For manuscripts utilizing custom algorithms or software that are central to the research but not yet described in published literature, software must be made available to editors and reviewers. We strongly encourage code deposition in a community repository (e.g. GitHub). See the Nature Portfolio guidelines for submitting code & software for further information.

Data

Policy information about availability of data

All manuscripts must include a data availability statement. This statement should provide the following information, where applicable:

- Accession codes, unique identifiers, or web links for publicly available datasets
- A description of any restrictions on data availability
- For clinical datasets or third party data, please ensure that the statement adheres to our policy

The datasets generated and analysed during the current study are available in the Gene Expression Omnibus (GEO) server with access number GSE232691.

Research involving human participants, their data, or biological material

Policy information about studies with human participants or human data. See also policy information about sex, gender (identity/presentation), and sexual orientation and race, ethnicity and racism.

Reporting on sex and gender	N/A
Reporting on race, ethnicity, or other socially relevant groupings	N/A
Population characteristics	N/A
Recruitment	N/A
Ethics oversight	N/A

Note that full information on the approval of the study protocol must also be provided in the manuscript.

Field-specific reporting

Please select the one below that is the best fit for your research. If you are not sure, read the appropriate sections before making your selection.

☒ Life sciences ☐ Behavioural & social sciences ☐ Ecological, evolutionary & environmental sciences

For a reference copy of the document with all sections, see nature.com/documents/nr-reporting-summary-flat.pdf

Life sciences study design

All studies must disclose on these points even when the disclosure is negative.

Sample size	For in vitro experiments, we used at least three biological replicates of each strain/condition. For SCID mice survival experiments, we performed two independent experiments with 6 mice/group, which resulted in n=12. For C57BL/6 infection experiments, we conducted two independent experiments with at least 6 mice/group. Statistical methods were not used to predetermine sample size. Sample size followed common standards in the field. The number of animals used for each experiments was estimated based on previous and pilot studies. Number of animals for each experimental group are described in Figure legends.
Data exclusions	For enumeration of CFUs in mice organs we routinely plate several dilutions and at least two technical replicates. However, eventually, some plates are contaminated with unrelated bacteria/fungi, and these contaminated plates are discarded for the enumeration of organ CFU burden.
Replication	In vitro experiments were performed at least in triplicate. Mice experiments were performed at least twice. In all cases, all attempts of replication were succesful.
Randomization	Mice were randomized into different experimental groups prior to bacterial infections
Blinding	Animal monitoring, sample collection and data analysis were not blinded since it is mandatory to identify the specific strain/condition tested to reach experimental conclussions.

Reporting for specific materials, systems and methods

We require information from authors about some types of materials, experimental systems and methods used in many studies. Here, indicate whether each material, system or method listed is relevant to your study. If you are not sure if a list item applies to your research, read the appropriate section before selecting a response.

Materials & experimental systems

n/a	Involved in the study	
☒	☐	Antibodies
☒	☐	Eukaryotic cell lines
☒	☐	Palaeontology and archaeology
☐	☒	Animals and other organisms
☒	☐	Clinical data
☒	☐	Dual use research of concern
☒	☐	Plants

Methods

n/a	Involved in the study	
☒	☐	ChIP-seq
☒	☐	Flow cytometry
☒	☐	MRI-based neuroimaging

Animals and other research organisms

Policy information about studies involving animals; ARRIVE guidelines recommended for reporting animal research, and Sex and Gender in Research

Laboratory animals	Mice strains used in the study were SCID and C57BL/6 purchased from ENVIGO. All were female mice of 6 weeks of age. Mouse experimentation and breeding were done in a SPF-facility at 20–24°C, 50–70% humidity, and a light-dark cycle of 12 h
Wild animals	No wild animals were used in the study
Reporting on sex	We routinely work with female mice
Field-collected samples	No field collected samples were used in this study
Ethics oversight	Ethics Committee for Animal Experiments from the University of Zaragoza (Project licenses: PI 31/21 and PI 43/23)

Note that full information on the approval of the study protocol must also be provided in the manuscript.

Plants

Seed stocks	N/A
Novel plant genotypes	N/A
Authentication	N/A
